# Extracellular vesicles in the treatment of oxidative stress injury: global research status and trends

**DOI:** 10.3389/fmolb.2023.1273113

**Published:** 2024-02-15

**Authors:** Wenwen Zhang, Bin Gan, Tingyu Wang, Xiangjie Yang, Yuanye Xue, Yuanqing Zhong, Xintong He, Xinsheng Peng, Yanfang Zhou, Xiaoyan Cheng

**Affiliations:** ^1^ Department of Pathophysiology, Guangdong Medical University, Dongguan, China; ^2^ The Third Affiliated Hospital of Guangdong Medical University, Fo Shan, China; ^3^ School of Public Health, Guangdong Medical University, Dongguan, China; ^4^ The Second Clinical Medical College, Guangdong Medical University, Dongguan, China; ^5^ School of Pharmacy, Guangdong Medical University, Dongguan, China; ^6^ Institute of Marine Medicine, Guangdong Medical University, Zhanjiang, China

**Keywords:** Exosomes, extracellular vesicles, oxidative Stress, bibliometrics, cluster analysis

## Abstract

**Objective:** The aim of this study was to conduct a bibliometric analysis of the literature on “Extracellular Vesicles in the Treatment of Oxidative Stress Injury” and to reveal its current status, hot spots and trends.

**Methods:** The relevant literature was obtained from the Web of Science Core Collection (WoSCC) on 29 April 2023. We performed clustering and partnership analysis of authors, institutions, countries, references and keywords in the literature through CiteSpace software and the bibliometric online analysis platform and mapped the relevant knowledge maps.

**Results:** A total of 1,321 relevant publications were included in the bibliometric analysis, with the number of publications in this field increasing year by year. These included 944 “articles” and 377 “reviews”. The maximum number of publications published in China is 512, and the maximum number of highly cited publications published in the United States is 20. Based on CiteSpace, the country collaboration network map shows close and stable collaboration among high-productivity countries. Based on WoSCC, there are 1706 relevant research institutions and 119 highly cited elite institutions, among which Kaohsing Chang Gung Men Hosp has the most extensive influence. Studies related to “Extracellular Vesicles in the Treatment of Oxidative Stress Injury” have been published in 548 journals. The keywords of the publications show the main research areas and breakthroughs. Based on WoSCC, the keywords of the research area “Extracellular Vesicles in the Treatment of Oxidative Stress Injury” were found to be as follows: exosome(s), extracellular vesicle(s), oxidative stress, inflammation, mesenchymal stem cells, apoptosis, microRNA (miRNA), mitochondria, biomarker, autophagy, angiogenesis and Alzheimer’s disease. Analysis showed that “mesenchymal stem cells”, “microRNA”, “autophagy”, “histology” and “therapeutic” emerged as highly explosive keywords.

**Conclusion:** This study is the first to use visual software and data mining to assess the literature in the field of “Extracellular Vesicles in the Treatment of Oxidative Stress Injury”. The research history, research status and direction in this field provide a theoretical basis for its scientific research.

## 1 Introduction

Extracellular vesicles (EVs) are membranous vesicles released by a variety of cells under different physiological and pathophysiological conditions. They can be detected in body fluids, including plasma, saliva, amniotic fluid and breast milk, and contain proteins, lipids and RNA representing host cells (Kalra et al., 2012). EVs are categorized according to their diameters, and the most numerous are small EVs, such as exosomes, small ectosomes (Mathieu et al., 2021), ciliary exosomes, small ectosomes (Mathieu et al., 2021), and ciliary ectosomes (Cao et al., 2015), etc. The number of medium-sized EVs with diameters of 200–800 nm is less than that of all EVs, such as microvesicles, FDC-derived iccosomes, T-cell microvilli particles (Kim et al., 2018), and elongated neutrophil-derived structures (Marki et al., 2021), etc. while the number of large EVs with diameters ≥1 μm is less. EVs with diameters ≥1 μm are fewer in number, such as apoptotic bodies, large oncosomes (Di Vizio et al., 2012), beaded apoptopodia (Atkin-Smith et al., 2019), migrasomes (Ma et al., 2015), exophers (Melentijevic et al., 2017), and en bloc-released virus clusters (Kerviel et al., 2021), etc. In addition, EVs also include vesicles produced by different cell death mechanisms, such as apoptosis, necrosis or focal death. Extracellular vesicles have many biological functions. They can quickly remove unnecessary molecules from cells, make cells mature and adapt to changes quickly, and activate blood coagulation. Extracellular vesicles, as units, can be transmitted between cells through surface proteins and encapsulated cargo molecules, thus regulating the functions of other cells. EVs are essential in various physiological and pathological aspects of the body. We can predict new biomarkers for disease treatment and drug monitoring, as well as new therapeutic and delivery tools, by making statistics based on the publication of recent articles and reviews in this field (Buzas, 2023).

Oxidative stress refers to the situation in which the level of oxygen and oxygen-derived free radicals exceeds the natural antioxidant defense capacity of cells (Bisht et al., 2017). The aggravation and prolongation of symptoms caused by oxidative stress is always a major problem in various common clinical diseases. The antioxidant activity of EVs has unique advantages in inhibiting oxidative damage and alleviating inflammatory reactions (Wang et al., 2020a). It is closely related to the regulation of oxidative stress damage in various systemic diseases.

At present, research on “Extracellular Vesicles in the Treatment of Oxidative Stress Injury” mainly focuses on apoptosis, autophagy, stem cells and miRNAs. The steady growth of research results in the above research directions shows that the exploration prospects are broad.

Bibliometrics combines mathematical statistics and literature. It uses quantification to explore the structural characteristics and hot trends of the discipline (Dong et al., 2022). At present, there is no bibliometric publication on the quantitative study of extracellular vesicles in the treatment of oxidative stress injury. Therefore, this paper uses bibliometrics to systematically review the research in this field and quantitatively related literature, and at the same time, with the help of visual literature tools, the research hotspots and frontiers in this research field are analyzed. Compared with a literature review, this study is a new attempt to review and visualize the research field on a large time scale from a global perspective to help relevant scholars understand the research status and trends in the world in time and provide certain guidance for researchers, scientific research and policy making (Liu et al., 2023).

## 2 Methods

### 2.1 Data source

The data were obtained from the Web of Science core of Clarivate (https://clarivate.com/) on 29 April 2023. The retrieval link is https://www.webofscience.com/wos/woscc/summary/cbffeb5d-278b-47a0-97e8-20438205b8a0-862ebd16/relevance/1. A total of 1,321 papers were retrieved, including 40 highly cited papers, 377 review papers, 31 online published papers and 943 open access papers. Topic terms (search title, abstract, author keywords and Keywords Plus) are used in the WoSCC database. The time range and paper form of the search are not limited. The search was limited to publications written in English. The search terms included “EVs”, “exosomes” and “oxidative stress”. All electronic searches were conducted on 29 April 2023. The literature was read independently by three reviewers. First, the literature was screened according to the title and abstract of the article and then screened again according to the inclusion and exclusion criteria. Included is any article or review related to the topic “Extracellular Vesicles in the Treatment of Oxidative Stress Injury”. After screening, if there was any dispute over the 1,321 selected publications, the final decision was discussed and voted on by the three evaluators again.

### 2.2 Data screening and processing

The retrieved journal publications on “Extracellular Vesicles in the Treatment of Oxidative Stress Injury” were imported into Note Express software and screened by three evaluators, and 1,321 papers were finally included. Document data exported from WoSCC are saved in “plain text file” and “tab delimited file” formats. Three researchers reviewed the selected articles and then imported them into CiteSpace software and OALM. Through the online analysis platform of bibliometrics (OALM) and CiteSpace, 1,321 publications on “Extracellular Vesicles in the Treatment of Oxidative Stress Injury” were comprehensively analyzed by number, country, journal, author, and keywords, and a visual graph was generated, in which each node represents different meanings, such as country, author, and journal. The size of the node reflects frequency and importance, and the color shade in the CiteSpace graph indicates year or different clusters. The nodes in the graph represent different meanings, such as country, author, and journal. The size of the nodes reflects the frequency and importance, and the color shades in the CiteSpace graph indicate the year or different clusters. We used Excel software to process the trend of the number of documents analyzed by the Online Analysis Platform for Bibliometrics (OALM), which was presented in the form of bar charts, histograms and pie charts. To prevent the cocitation network from being too complex, this paper adopts the Pathfinder Algorithm (PFA) to simplify the network by removing the edges that violate the triangular inequality and accurately extracting the key structure of the network.

### 2.3 Document measurement index

The literature measures used in this paper mainly include the following four indexes: frequency, centrality, detonation degree and clustering. Frequency indicates that the selected publications appear in different classifications, and the research hotspots and trends of 1,321 publications showing “Extracellular Vesicles in the Treatment of Oxidative Stress Injury” are classified by years and countries so that readers can clearly understand the history of this research field and find future exploration directions. Centrality is an important index of document clustering and a central measure of the degree of control of network nodes over resources in document metrology graphs, which mainly measures the role of each node in a specific network graph. If the centrality of a node is higher, the more the node appears on the shortest path in the network graph, the greater the possibility that other nodes will establish co-occurrence relationships with it, and the greater the influence and importance of the node in the network graph. Nodes with centrality exceeding 0.1 are called critical nodes (Liu et al., 2023); “Detonation” means causing something to cause a sensation. “Detonation degree” means the strength of the sensational effect. Key words of publications, the detonation degree of publications and authors indicate that this index is highly concerned and belongs to hot content within the corresponding age span. Clustering refers to grouping physical or abstract objects into multiple classes composed of similar objects. The goal is to collect data on the basis of similarity to classify and measure the similarity between different data sources and classify data sources into different clusters.

## 3 Result

### 3.1 Quantitative analysis

**FIGURE 1:**
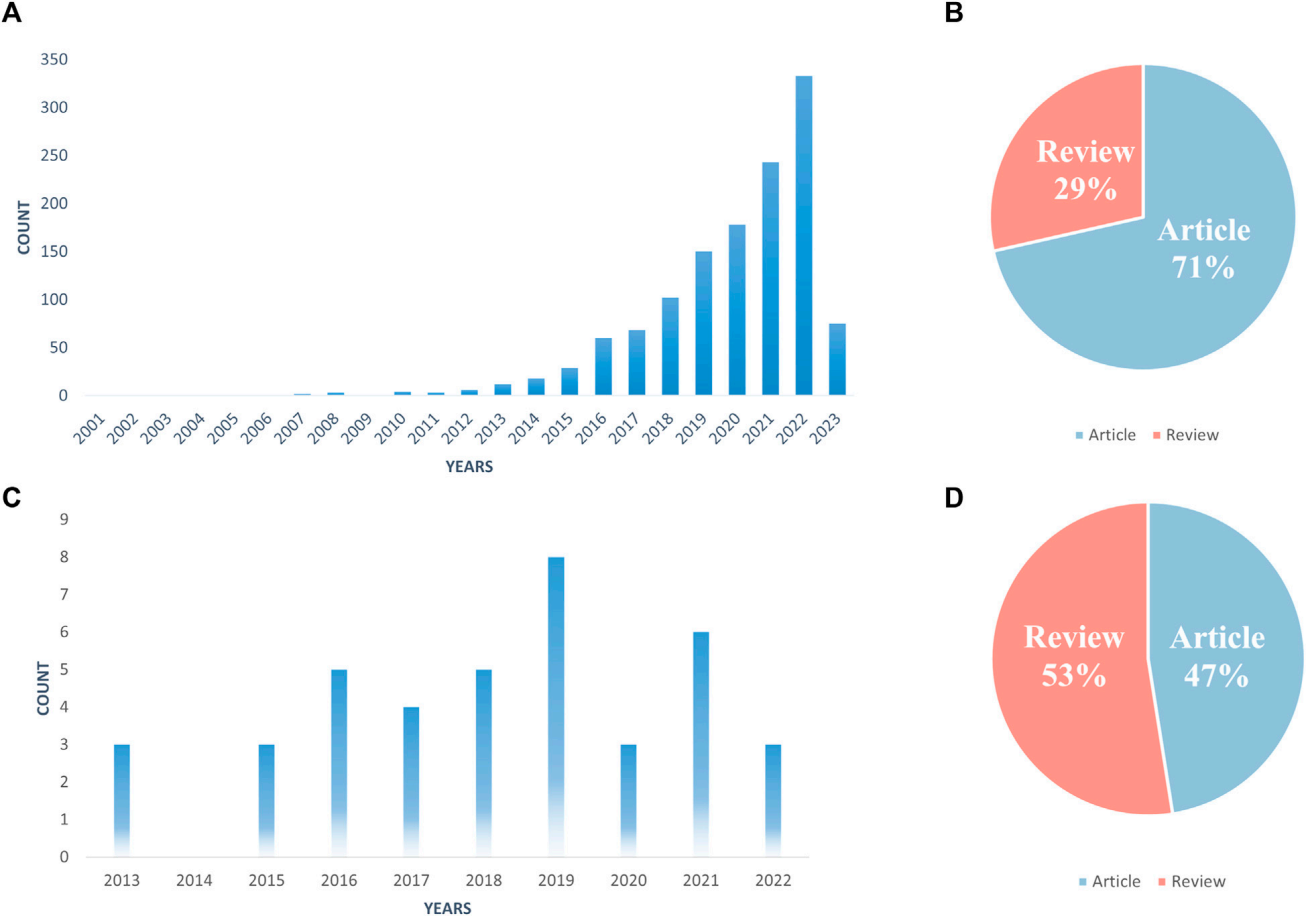
Number of publications per year **(A, C)**; literature type **(B, D)**; **(A, B)** All related literature; **(C, D)** Highly cited essentials).

According to the statistical graph derived from the search strategy (Figure 1A), the number of papers published on “Extracellular Vesicles in the Treatment of Oxidative Stress Injury” is generally on the rise, which indicates that this research has gradually been emphasized, and the research value and application fields are becoming increasingly extensive. The total number of studies on “Extracellular Vesicles in the Treatment of Oxidative Stress Injury” was 1,321, including 944″articles” and 377″reviews” (Figure 1B). From the point of view of the growth rate of the number of papers published each year, the whole period can be divided into three parts: the first phase (2001–2006), during which few scholars studied the interactions between exosomes and oxidative stress, and there were only a few papers, and this kind of research was still in the initial stage of germination; the second phase (2007–2015), during which the research on the treatment of oxidative stress-related disorders with exosomes entered the initial period of exploration, and the average annual publication rate was 1,040 articles and 1,377 reviews (Figure 1B). In the second phase (2007–2015), research on exosomes for oxidative stress-related diseases entered the initial exploration period, with an average of 8.67 articles per year; in the third phase (2016–2023), the number of papers in this direction exploded and entered a prosperous period, and the number of papers is currently at its peak in 2022, with 333 articles. 2023, the number of articles is 75 according to the statistics of “Extracellular Vesicles in the Treatment of Oxidative Stress Injury”. Based on the growing trend of research papers related to “Extracellular Vesicles in the Treatment of Oxidative Stress Injury”, it is predicted that this type of research will reach a new peak after the end of 2023.

Price’s Law describes the pattern of change in the volume of literature in a particular category over time and predicts future trends. Based on Price’s law, we calculate the equation of the exponential curve for the third stage (2016–2023) as y = 4E-284e^0.3256x^. The simulated curve fits well with the annual growth trend of the literature with a high coefficient of determination (R^2^ = 0.9922). Based on the exponential curve equation, we can predict that the annual growth of articles will continue in the coming years. In addition, we selected 40 highly cited papers in this research direction, which we call “Highly Cited Essentials” (Figure 1C). Highly cited papers have gradually appeared since 2013, and the highest number of highly cited quintessences was published in 2019, with 8 papers. However, half of the highly cited essences belong to reviews, with 21 out of 40 (Figure 1D), indicating that this research area has great explorability and promising prospects for development.

### 3.2 Country analysis

**FIGURE 2:**
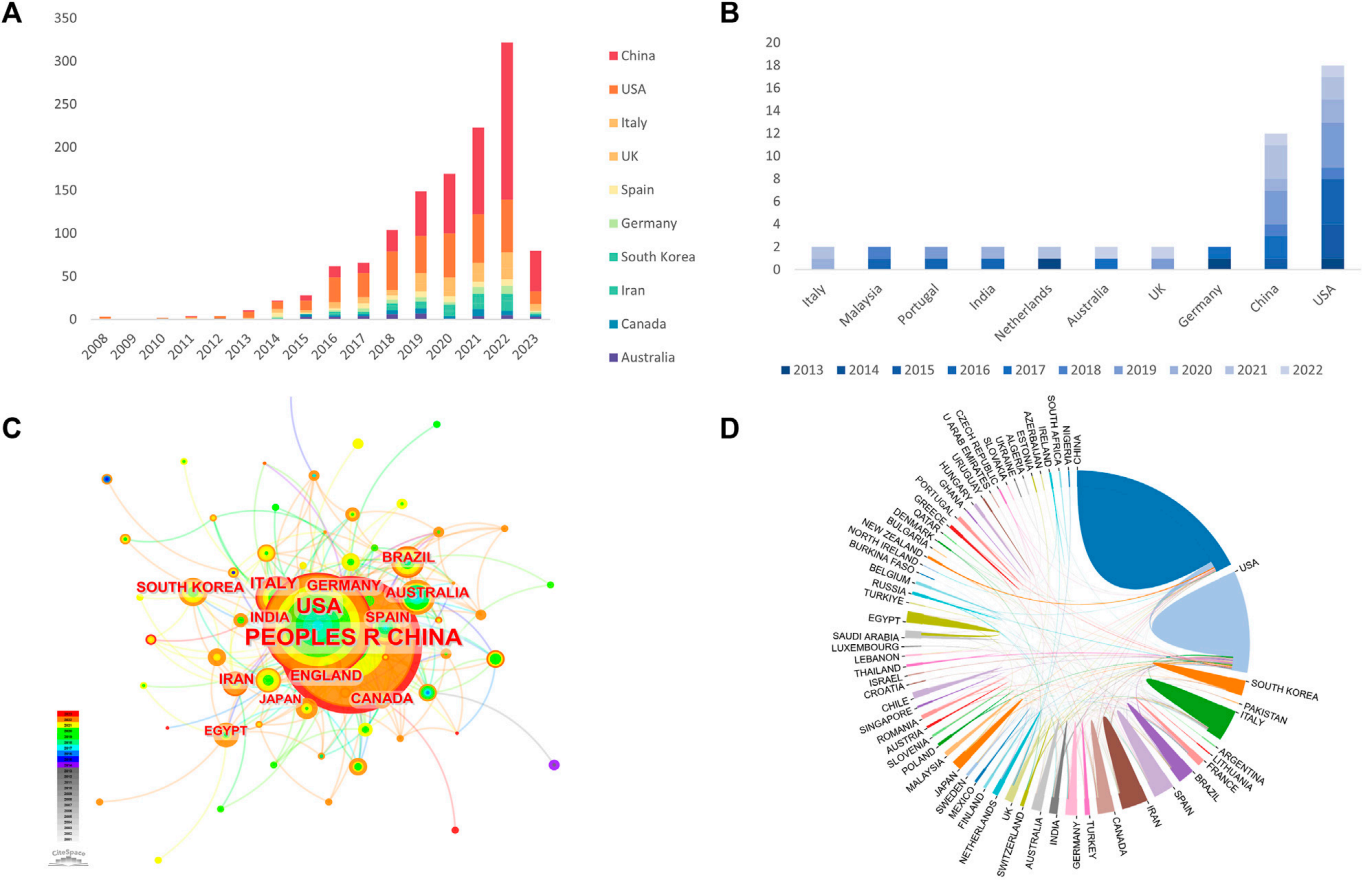
Country of Related Literature **(A, B)**; Country Cooperation **(C, D)**; **(A, C, D)** All relevant literature; **(B)** Highly cited essence).

The statistics on the number of papers published in the total number of countries found that, based on the core set of Web of Science, the research on “Extracellular Vesicles in the Treatment of Oxidative Stress Injury” involves 10 countries/regions, and the countries/regions that produced the most highly cited articles have slightly changed. China published the most articles with 512 (Figure 2A), and the United States published the most highly cited articles with 20 (Figure 2B). Based on CiteSpace, a map of national collaboration networks shows close and stable collaborations between high-productivity countries (Figure 2C), with the United States, China, Italy, and the United Kingdom collaborating more with institutions in other countries, and the United States collaborating the most extensively and conducting the most advanced research related to “Extracellular Vesicles in the Treatment of Oxidative Stress Injury” (Figure 2D). The United States has the most extensive collaboration and is the leader in conducting research on “Extracellular Vesicles in the Treatment of Oxidative Stress Injury” (Figure 2D), and China’s research development in this area has started gradually since 2013, while more than half of the other countries have fewer interactions with other countries (Figure 2D).

### 3.3 Institutional analysis

**FIGURE 3:**
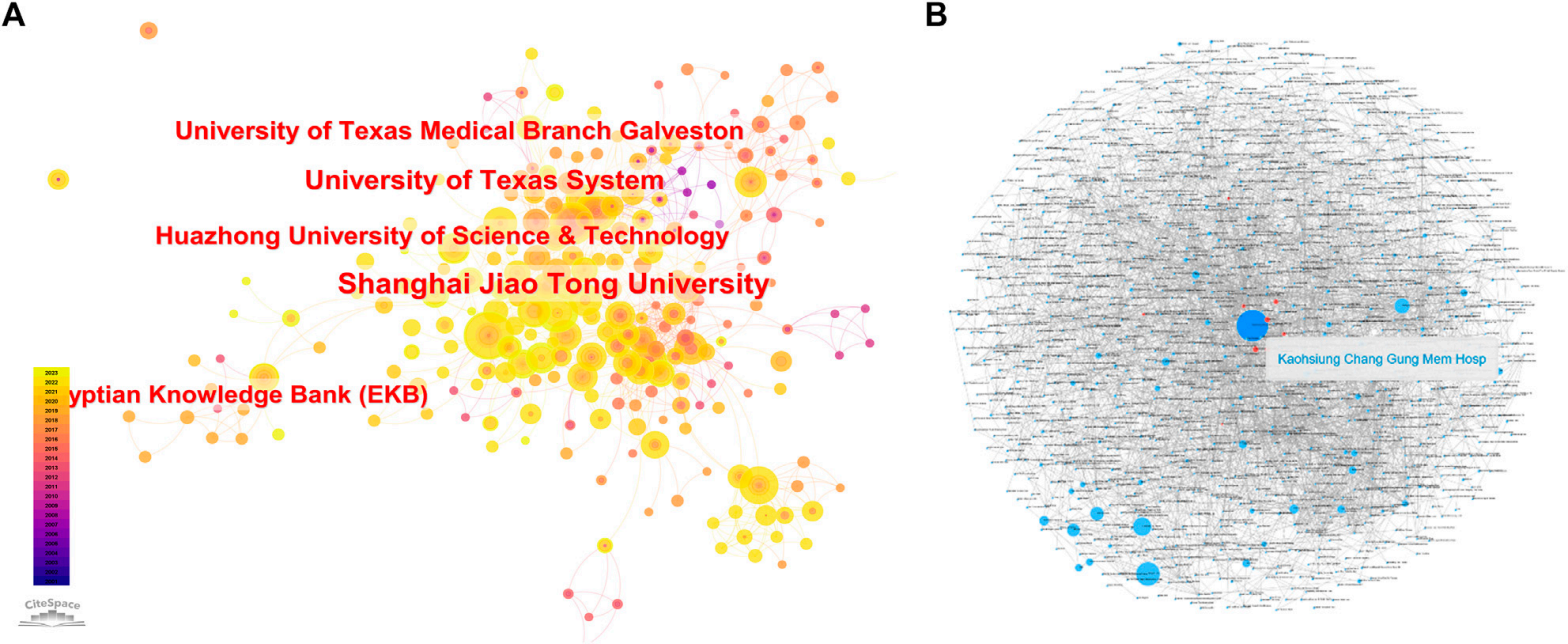
Institutions of relevant literature **(A)**. Cooperation between institutions **(B)**. **(B)** Each small blue dot represents an organization link represents collaboration. The larger the blue dot, the more collaboration. Deep Blue Dot is the agency that cooperates the most. Red Dot is the agency that cooperates with this agency.

Based on the WoSCC, there are 1,706 related research institutions and 119 highly cited elite institutions. Among them, Kaohsing Chang Gung Men Hosp had the widest reach (Figure 3B), with 48 publications, and this institution had the most cited articles (477). The four institutions with the highest average number of citations were ASTAR, Netherlands Heart Inst ICIN, NUS, and First Peoples Hosp Lianyungang, with averages of 92, 92, 92, and 65 citations, respectively. Shanghai Jiao Tong University had the highest number of papers (Table 1) at 68, but the total number of citations was slightly lower compared to Kaohsing Chang Gung Men Hosp at 292 (Table 1). The institutions of publications were filtered and visualized based on CiteSpace, and a collaboration network was constructed based on the number of publications and relationships of each institution (Figure 3A). The diagram of the institutional collaboration network shows that there is close collaboration among research institutions, dominated by individual universities, e.g., Shanghai Jiao Tong University, University of Texas, Harvard University, and Johns Hopkins University.

**TABLE 1:** Top 10 research institutions.

Organization (country)	Article Counts	Total number of citations	Average citations	Total number of first authors	Number of first author citations	Average number of first author citations
Kaohsiung Chang Gung Mem Hosp (Taiwan, China)	48	477	9.94	6	50	8.33
Jiangsu Univ (China)	20	434	21.70	8	129	16.13
Shanghai Jiao Tong Univ (China)	68	292	4.29	29	82	2.83
Univ Med Ctr Utrecht (Netherlands)	5	279	55.80	3	95	31.67
ASTAR (Singapore)	2	184	92.00	0	0	0.00
Univ Valencia (Spain)	16	165	10.31	6	17	2.83
Univ Texas Med Branch (the United States)	33	148	4.48	19	90	4.74
Natl Univ Singapore (Singapore)	4	134	33.50	2	39	19.50
Univ Pittsburgh (the United States)	18	132	7.33	4	3	0.75
Univ Calif San Francisco (the United States)	14	111	7.93	3	29	9.67

### 3.4 Authors’ analysis

Based on CiteSpace, there are 525 authors in all related studies (Figure 1A). Among them, Menon, Ramkumar, Kumar, Santosh and Kododela, Sunitha have the largest nodes with the highest cumulative number of publications. The number of publications on “Extracellular Vesicles in the Treatment of Oxidative Stress Injury” was 20, 13 and 11, respectively. The authors’ collaborative network diagram (Figure 1B) shows that there is a collaborative relationship between the high-producing authors, forming a stable research team. In all the studies, Menon and Ramkumar were also authors who collaborated extensively with other scholars. In addition, we observed close collaboration between multiple authors. For example, Liu YT has close collaboration with Hamrick MW, Zhang K, and Zhou YL; Chen ZQ has active collaboration with Wang YX. Among the many authors who produced highly cited essences (Figure 1C), most of the scholars who worked closely with each other belonged to the same research institution, authors who belonged to different institutions collaborated less with each other, and Zhang, X collaborated more closely with a large number of scholars from both institutions.

### 3.5 Publication analysis

**FIGURE 4:**
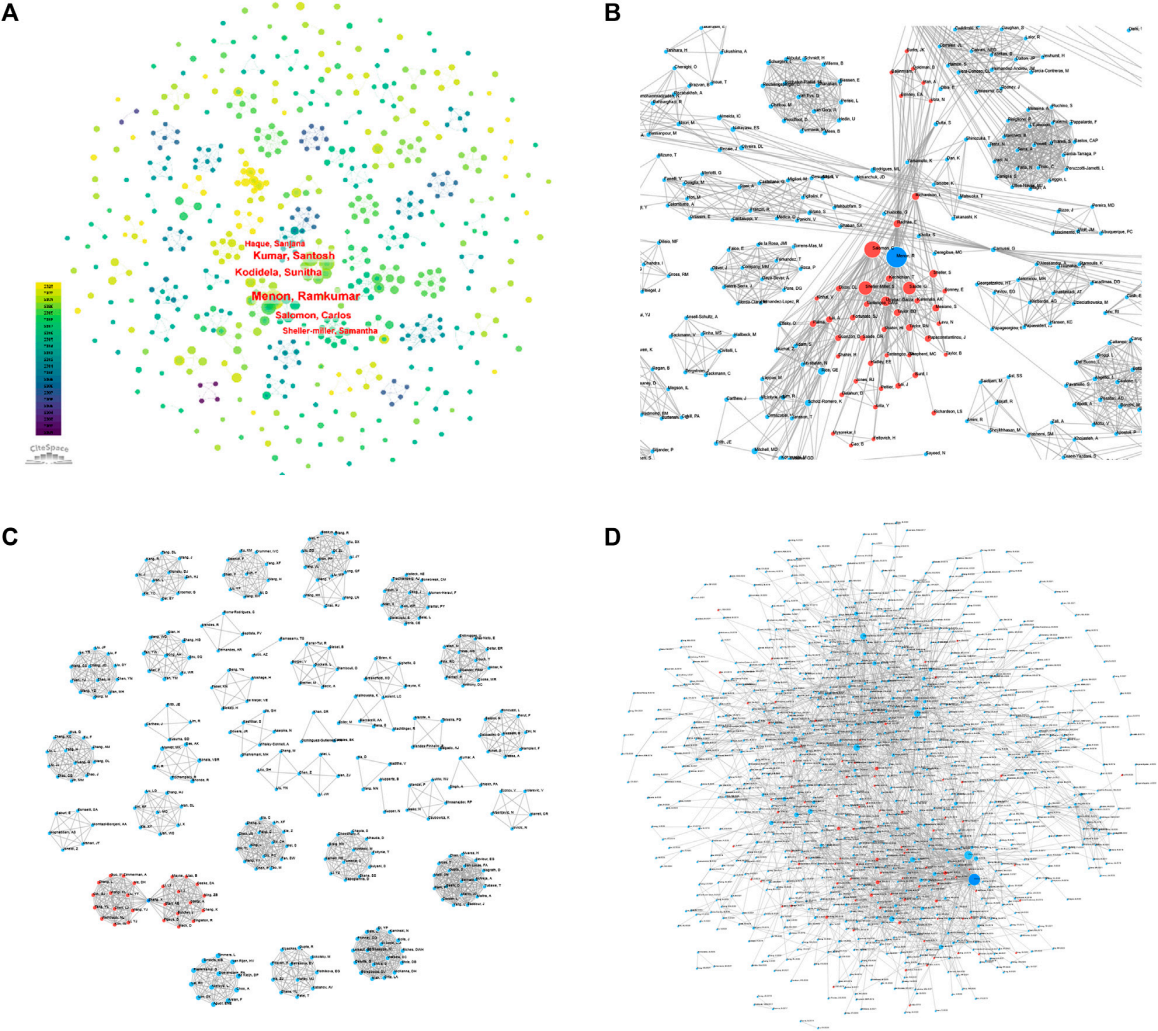
Collaborative relationship between authors of related literature **(A, B, C)**. **(A, B)** All relevant literature; **(C)** Highly cited essentials; **(B, C)** Each small blue dot represents an author link for collaboration; The bigger the blue dot, the more collaboration; The deep blue dot is the author who collaborates the most; The red dot is the author who collaborates with the author). Citation Relation Network of Journals and Related Documents **(D)**. **(D)**: All relevant literature; Each small blue dot represents an article. Link represents a reference. The larger the blue dot, the more references. The deep blue dot is the most cited article, and the red dot is the article that quoted the article.)

**FIGURE 5:**
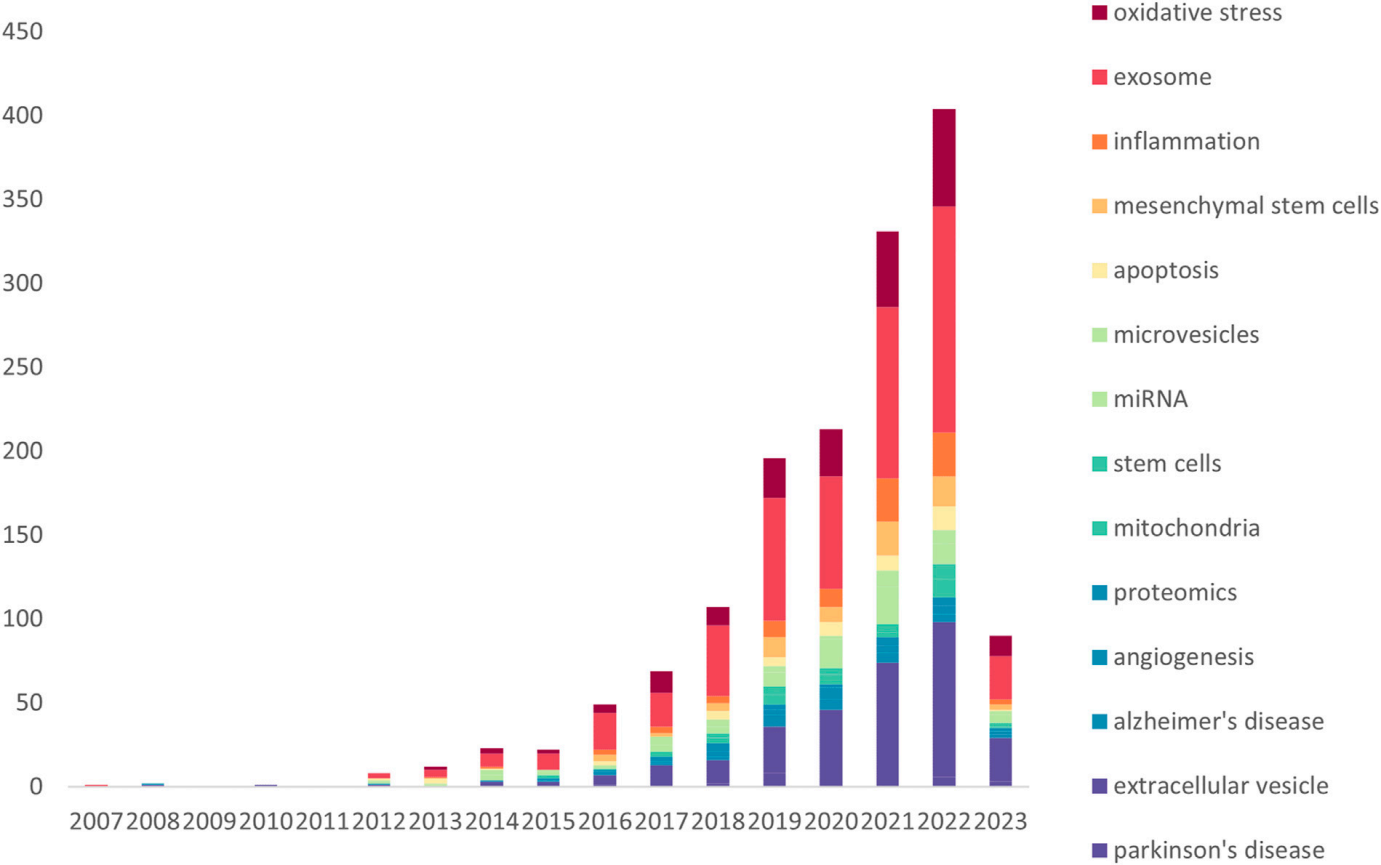
A: Annual number of commonly used keywords.

Based on WoSCC (Table 2), studies related to “Extracellular Vesicles in the Treatment of Oxidative Stress Injury” have been published in 548 journals. The largest number of publications is in the *International Journal of Molecular Sciences* (59), followed by *Stem Cell Research & Therapy* (38), *Oxidative Medicine and Cellular Longevity* (38) and *Cells* (31 articles). The highest total citations were for *Stem Cell Research & Therapy* with 206 citations, and the highest average citations were for *Stem Cell Research* with 92 citations. Among the top 10 journals with the highest total citations, the journal with the highest impact factor was *Free Radical Biology and Medicine* (IF = 7.4). As seen from the article citation relationship network diagram (Figure 5A), the articles that are more closely related to each other and are cross-cited have the highest number of citations in Arslan, F et al., 2013, “Mesenchymal stem cell-derived exosomes increase ATP levels, decrease oxidative stress and activate PI3K/Akt pathway to enhance myocardial viability and prevent adverse remodeling after myocardial ischemia/reperfusion injury” (Figure 4D), but the year of its publication is more advanced, and the highly cited essences of the last 3 years are Zhao, RT-2022 and Zhao, M- 2021, respectively, published in the Journal of the International Academy of Sciences. RT-2022 and Zhao, M-2021, published in the Journal of Controlled Release (IF = 10.8) and *ACS Nano* (IF = 17.1), respectively, and both of them are researching “Extracellular Vesicles in the Treatment of Oxidative Stress Injury”, which indicates that this research direction has promising prospects for exploration. This indicates that this research direction is promising.

**TABLE 2:** Introduction to publications.

Journal	Article Counts	Total citations	Average citations	If
Stem Cell Research & Therapy	38	206	5.42	7.5
PLoS One	18	168	9.88	3.7
Stem Cell Research	1	92	92.00	1.2
International Journal of Molecular Sciences	59	72	1.22	5.6
Journal of Cellular and Molecular Medicine	13	70	5.38	5.3
Scientific Reports	20	66	3.03	4.6
International Journal of Cardiology	3	63	31.00	3.5
Oxidative Medicine and Cellular Longevity	38	62	1.63	7.31
Free Radical Biology and Medicine	38	62	1.63	7.4
Stem Cells International	12	57	4.75	4.3

### 3.6 Keyword analysis

#### 3.6.1 Word frequency analysis

The keywords of a publication show its main research areas and breakthroughs, which allow scholars to accurately find the target group of literature when searching the literature. The changes in keywords in the research field also represent the changes in the hotspots of exploration and the depth of the research results in the research field, which is an important representation of the publication only after the title. Based on WoSCC, the keywords in the research field of “Extracellular Vesicles in the Treatment of Oxidative Stress Injury” were found to be the following by analyzing the word frequency of the keywords over the years (Figure 5A; Table 3): exosome(s), extracellular vesicle(s), oxidative stress, oxidative stress, and other keywords. The key words in this research area are as follows: exosome(s), extracellular vesicle(s), oxidative stress, inflammation, mesenchymal stem cells, apoptosis, microRNA (miRNA), mitochondria, biomarker, autophagy, angiogenesis, and Alzheimer’s disease, where the top five keyword word frequencies are exosome(s) (513), extracellular vesicle(s) (302), oxidative stress (203), stem cells (mesenchymal stem cells) (106) and inflammation (89). As seen, while studying “Extracellular Vesicles in the Treatment of Oxidative Stress Injury”, numerous scholars also explore exosomes and ex vesicles for inflammation and apoptosis at the same time, and in general, oxidative stress and inflammation and apoptosis are intertwined and cocausing diseases. From 2015, stem cell exosomes gradually began to be studied, and until 2019, the number of publications on “Extracellular Vesicles in the Treatment of Oxidative Stress Injury” gradually increased, peaking at 27 in 2022, indicating that this research field is booming and has a very bright future.

**TABLE 3:** Frequency of commonly used keywords.

Rank	Keywords	Count	Year	Rank	Keywords	Count	Year
1	oxidative stress	657	2001	26	gene expression	43	2014
2	extracellular vesicles	410	2014	27	pathway	41	2014
3	exosome	242	2008	28	disease	40	2014
4	expression	170	2007	29	myocardial infarction	40	2016
5	mesenchymal stem cells	142	2016	30	differentiation	39	2013
6	cells	107	2014	31	protein	39	2007
7	apoptosis	101	2010	32	inhibition	37	2017
8	activation	92	2007	33	secretion	37	2016
9	stem cells	89	2015	34	autophagy	36	2014
10	inflammation	89	2016	35	biomarkers	35	2013
11	microvesicles	70	2013	36	bone marrow	35	2014
12	stromal cells	76	2015	37	parkinsons disease	34	2008
13	*in vitro*	76	2012	38	repair	34	2018
14	injury	69	2017	39	growth	30	2005
15	mechanisms	63	2013	40	pathogenesis	30	2010
16	proliferation	58	2016	41	ischemia‒reperfusion injury	29	2016
17	microRNAs	57	2016	42	adipose tissue	28	2007
18	Alzheimer’s disease	55	2010	43	transplantation	28	2019
19	angiogenesis	55	2017	44	macrophages	27	2019
20	Oxidative stress	53	2016	45	nf kappa b	27	2012
21	biogenesis	51	2010	46	stress	27	2008
22	brain	50	2015	47	extracellular vesicle	26	2018
23	dysfunction	50	2017	48	mechanism	26	2007
24	endothelial cells	49	2012	49	damage	25	2019
25	therapy	44	2015	50	delivery	25	2020

#### 3.6.2 Cluster analysis

**FIGURE 6:**
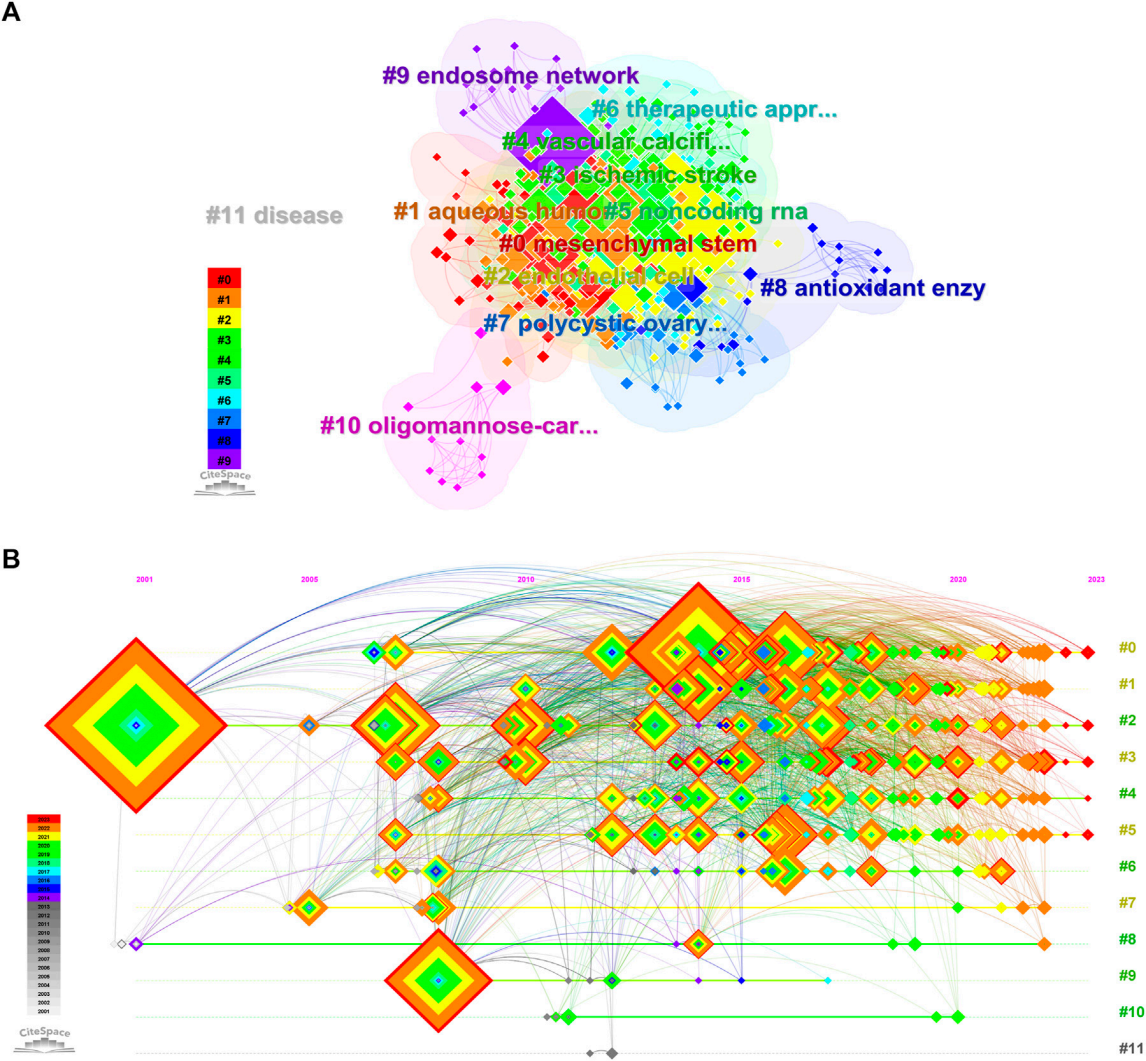
Clustering view of related literature keywords **(A)**; Keyword Clustering Timeline View of Related Literature **(B)**, **(A, B)** All relevant literature).

The keywords of the publication are clustered by the criterion of “the same in the same category and different in the different categories”, and the results of the cluster analysis are labeled by the log-likelihood ratio (LLR) method to explore and excavate the potential differences and connections of the keywords and to reflect the hotspots of the research direction and the future research direction. The “Q” value in the cluster analysis timeline graph (Figure 6) is used to evaluate the performance of the clustering network, and the “S” value is used to measure the homogeneity of the cluster members. Q > 0.3 and S > 0.5 indicate that the structure of the obtained clustering network is significantly convincing and that the clustering results are reasonable, respectively. The “Q” value of 0.3769 and the “S” value of 0.6712 in Figure 6A, B, respectively, indicate that these clustering graphs are reasonable and that the clustering results are reasonable. indicating that these clustering diagrams are reasonable and informative. In the study of “Extracellular Vesicles in the Treatment of Oxidative Stress Injury”, there are 12 meaningful clusters (Table 4), and there are several overlapping clusters in the clustering diagram, indicating that the research is highly relevant, among which stem cells contain the most keywords, indicating that stem cells play an important role in the treatment of oxidative stress injury and have the potential to play an important role in the treatment of oxidative stress injury and have the potential to play a significant role in the treatment of oxidative stress. and play an important role in the treatment of oxidative stress injury, with numerous points that can be mined and explored.

**TABLE 4:** Keyword co-occurrence network clustering table.

Cluster ID	Size	Mean (Year)	Label (LLR)
0	94	2017	mesenchymal stem (1,426.66, 1.0E-4); cardiovascular disease (948.07, 1.0E-4); stromal cell-derived extracellular vesicle (677.09, 1.0E-4); chronic kidney disease (574.2, 1.0E-4); admsc-derived exosome (512.23, 1.0E-4)
1	85	2018	aqueous humor (480.75, 1.0E-4); irradiation-induced hematopoietic system injury (339.42, 1.0E-4); mouse serum (339.42, 1.0E-4); total body (339.42, 1.0E-4); systemic environment (339.42, 1.0E-4)
2	69	2013	endothelial cell (523.22, 1.0E-4); mesenchymal stem cell (477.68, 1.0E-4); stem cell (447.09, 1.0E-4); therapeutic potential (412.44, 1.0E-4); human umbilical cord (395.42, 1.0E-4)
3	69	2017	ischemic stroke (655.71, 1.0E-4); protein aggregation (607.18, 1.0E-4); neural stem (583.6, 1.0E-4); parkinsons disease (580.16, 1.0E-4); neurotrophic factor (555.31, 1.0E-4)
4	64	2016	vascular calcification (803.61, 1.0E-4); secretory protein (378.95, 1.0E-4); vascular research (378.95, 1.0E-4); age-associated stroke (372.63, 1.0E-4); human parturition (366.31, 1.0E-4)
5	47	2017	noncoding rna (561.54, 1.0E-4); vitro model (463.36, 1.0E-4); systematic mini review (395.83, 1.0E-4); mitochondrial inactivity (391.17, 1.0E-4); obesity-related cardiomyopathy (391.17, 1.0E-4)
6	39	2013	therapeutic approaches (435.39, 1.0E-4); myocardial injury (404.77, 1.0E-4); diabetic cardiomyopathy (332.73, 1.0E-4); 3t3-l1 adipocyte (320.6, 1.0E-4); dependent induction (320.6, 1.0E-4)
7	25	2011	polycystic ovary syndrome (372.15, 1.0E-4); comparative lipid peroxidation (221.73, 1.0E-4); proline content (221.73, 1.0E-4); antioxidant defense system (221.73, 1.0E-4); rice cultivar (221.73, 1.0E-4)
8	19	2007	antioxidant enzyme (114.5, 1.0E-4); growing rice seedling (114.5, 1.0E-4); lipid peroxidation superoxide anion generation (114.5, 1.0E-4); neuronal firing rate signal transduction (100.12, 1.0E-4); oligodendroglial exosome (100.12, 1.0E-4)
9	18	2012	endosome network (300.24, 1.0E-4); blood-borne macrophage-neural cell interaction (300.24, 1.0E-4); cell-based nanozyme brain delivery (300.24, 1.0E-4); pigmented cell (294.61, 1.0E-4); melanosome autophagy (294.61, 1.0E-4)
10	11	2012	oligomannose-carrying glycoprotein act (52, 1.0E-4); glia-derived exosome (52, 1.0E-4); oligomannose-binding lectin (52, 1.0E-4); neurite outgrowth (52, 1.0E-4); neuronal survival (52, 1.0E-4)
11	8	2012	disease (21.06, 1.0E-4); retinal function (21.06, 1.0E-4); alpha-crystallin (21.06, 1.0E-4); novel role (21.06, 1.0E-4); extracellular vesicle (0.39, 1.0)

#### 3.6.3 Detonation level analysis

**FIGURE 7:**
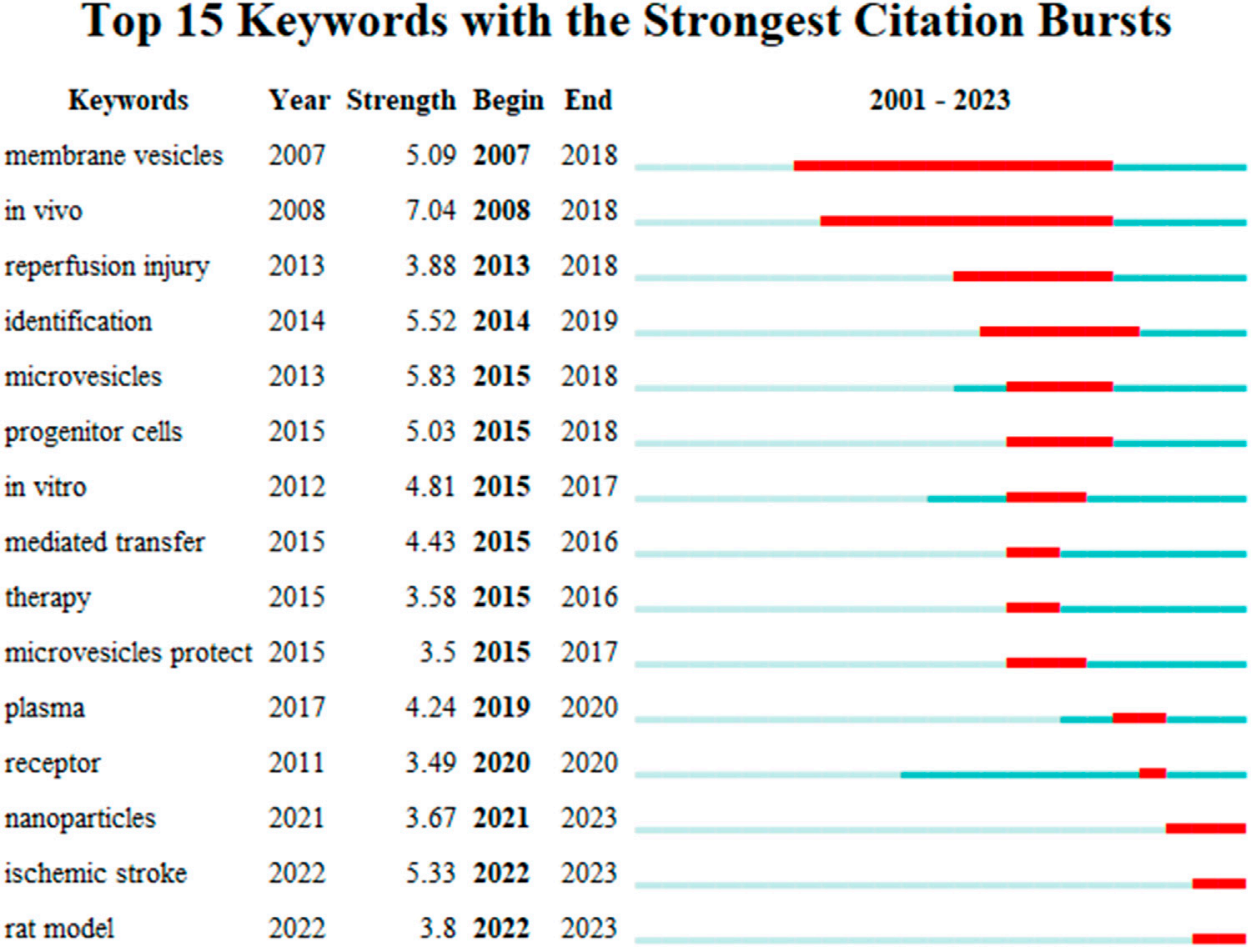
The detonation degree γ [0, 1] = 0.9, Minimum Duration = 1.

Keyword detonation refers to the frequency of simultaneous appearances and the degree of sudden change of a keyword in a period of time and is mainly used to study hotspots in a specific period of time. In this study, CiteSpace identified 15 keywords with strong bursts (Figure 7). The citation bursts for the keywords appeared as early as 2007 and as late as 2022. The keyword with the strongest outburst (intensity = 7.04) was “*in vivo*”, with outbursts from 2007 to 2018. The keyword with the second strongest outbreak (intensity = 5.09) was “membrane vesicles”, with outbreaks from 2008 to 2018. Overall, the 15 keywords had outbreak intensities ranging from 3.49 to 7.04, with endurance ranging from 1 to 11 years, spanning a range of years.

## 4 Discussion

### 4.1 Basic information

Extracellular vesicles are a means of long-distance cell-to-cell communication in organisms and are mainly categorized as exosomes, extracellular vesicles, and apoptotic vesicles with a double-membrane structure. Exosomes are small membrane vesicles containing complex RNA and proteins with a diameter of 40–160 nm (average 100 nm). Exosomes can be produced in cell cultures or in the supernatants of body fluids, e.g., blood, saliva, urine, breast milk, cerebrospinal fluid, bile, and lymph (Caplan and Correa, 2011; Morrison et al., 2017; Ogisu et al., 2020). Exosomes carry a variety of genetic material, including microRNAs (miRNAs) and mRNAs, which harbor the biological functions of parental cells, possessing numerous functions, such as disease therapy and cellular communication (Xia et al., 2019). Compared to the parental cells of exosomes, exosomes have many advantages, such as low immunogenicity, high viability, and ease of crossing biological barriers, making them a safe and effective alternative to cell therapy (Wang et al., 2020b).

Oxidative stress (OS) is a state in which there is an imbalance between oxidative and antioxidant effects in the body, leading to inflammatory infiltration of neutrophils, increased secretion of proteases, and production of large amounts of oxidized intermediates. Oxidative stress is a negative effect produced by free radicals in the body and is an important pathogenic factor of many diseases. Today, the field of oxidative stress research includes chemistry, biochemistry, cell biology, physiology and pathophysiology, all the way to medicine, health and disease research (Sies, 2015).

Research on “exosomes” first appeared in 1970, and “oxidative stress” was first proposed in 1960. Based on the WoSCC, we found that “Extracellular Vesicles in the Treatment of Oxidative Stress Injury” has been gradually explored by scholars since 2001. The research on “Interaction of Exosomes and Extracellular Vesicles with Oxidative Stress” is 30–40 years behind the research on “exosomes and extracellular vesicles” and “oxidative stress”, which indicates that it is extremely difficult to determine the interaction of each research direction. This suggests that it is extremely difficult to identify interactions between different research directions and that it is important to conduct bibliometric studies in each research area. Bibliometrics can visualize the development hotspots and exploration history of different research fields, which can enable scholars to find the intersection of different research fields or the future development trend of each research direction in the same field and the breakthrough point of interaction research.

### 4.2 Status of global research

This paper is the first bibliometric analysis of “Extracellular Vesicles in the Treatment of Oxidative Stress Injury” and explores the research hotspots and development trends based on the data information. From the frequency of articles published year by year, there are an increasing number of publications in this research field, which indicates that this research direction is receiving increasing attention and has broad development prospects. From the perspective of global research trends, the United States is the core country in this field of research, with an early start of research, a large number of publications, the most highly cited essences, and a large impact of the research content, which is a leading position in the world. Among the top five countries in the number of publications, China is the only developing country, with the largest number of articles on “Extracellular Vesicles in the Treatment of Oxidative Stress Injury” and the second highest number of citations after the United States, which indicates that China is steadily advancing in this field and seeking new breakthroughs with other countries in the world. Based on the institutional analysis of CiteSpace and WoSCC, Shanghai Jiao Tong University is the research institution with the largest number of publications. Kaohsing Chang Gung Men Hosp has the widest influence, and there are different degrees of cooperation among various institutions. The collaborative network represents the most active and representative research team in the world, which can provide scientific and technical support for other scholars to carry out related research. Reference for other scholars to conduct related research (Liu et al., 2023).

### 4.3 Hot topics and frontiers

**FIGURE 8:**
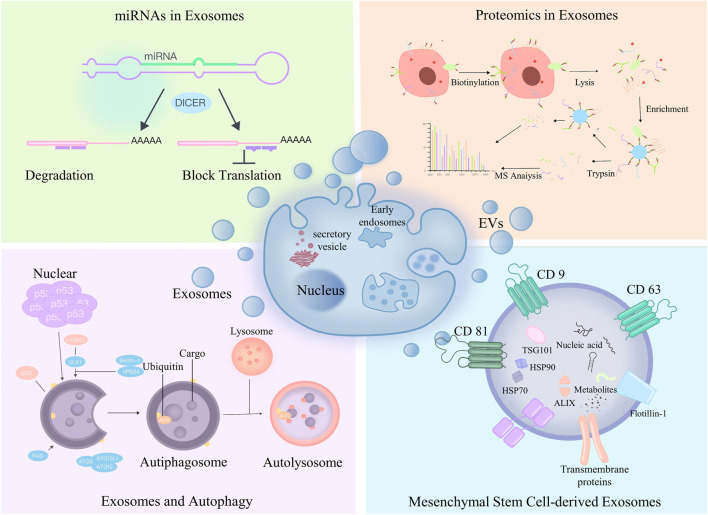
We found out four hot spots in exosomes at present by bibliometrics: Upper left corner: miRNA in exosomes; Upper right corner: exosomes are combined with proteomics; Lower left corner: exosomes and autophagy; Lower right corner: exosomes of mesenchymal stem cells.

The keywords of “Extracellular Vesicles in the Treatment of Oxidative Stress Injury” were visualized by CiteSpace and WoSCC, and it was found that the hot words were mainly “Parkinson’s disease”, “extracellular vesicle”, “Alzheimer’s disease”, “angiogenesis”, “proteomics”, “vesicle”, “vesicle”, “vesicle”, “vesicle”, “vesicle”, “vesicle”, “vesicle”, and “vesicle”, “proteomics”, “mitochondria”, “stem cells”, “miRNA”, “mitochondria”, “mitochondria”, and “mitochondria”. miRNA”, “microvesicles”, “apoptosis”, “mesenchymal stem cells”, “inflammation”, “exosome”, and “oxidative stress”. In addition to the hot words, we also analyzed the highly triggered keywords between different years by CiteSpace to quickly capture the distribution and evolution of hot words in the research field of “Extracellular Vesicles in the Treatment of Oxidative Stress Injury”. Based on the keyword clustering analysis and the analysis of highly triggered keywords, we conclude that the research on “extracellular vesicles in the treatment of oxidative stress injury” mainly focuses on the following aspects (Figure 8).

#### 4.3.1 Mesenchymal stem cell-derived exosomes

MSCs belong to a heterogeneous cell population of stromal cells that proliferate and differentiate *in vitro* as plastic-apposed cells (Friedenstein et al., 1974). MSCs have been shown to have various biological functions, including interacting with cells in the immune system for immunomodulation, inhibiting tumor necrosis factor (TNF), and upregulating IL-10 (Ding et al., 2015) to reduce inflammation, inhibit respiratory bursts and activate spontaneous apoptosis of neutrophils (Raffaghello et al., 2008). In recent years, most results have shown that MSC-derived exosomes and extracellular vesicles are fruitful in the treatment of oxidative stress injury. MSC-derived exosomes (MSC-Exos) have similar biological functions as MSCs, but MSC-Exos are more stable and less toxic and can largely avoid adverse events such as body-induced immune responses and capillary embolism, so researchers are actively exploring the strategy of MSC-Exos in the treatment of oxidative stress injury (Nojehdehi et al., 2018; Liu et al., 2020).

#### 4.3.2 miRNAs in exosomes

miRNA is currently the most studied substance in exosome content. miRNA contains the genetic material of many parental cells and is also the main source of the therapeutic effects of exosomes. In numerous publications, after exploratory research by scholars from various countries around the world, numerous exosomal miRNAs have been found to have good therapeutic effects on oxidative stress injury; for example, miR125a, miR-181b, miR-22-3p and miR-126 promote the repair of oxidative stress injury in the lungs (Chen et al., 2021; Zhao et al., 2022); miR-182-5p, miR-199a-3p, miR-214 and miR-223 treat oxidative stress injury in the heart (Qin et al., 2018); miR-215-5p, miR-424-5p, miR-31-3p, miR 193b-3p and miR-200b-3p promote the repair of oxidative damage in the nervous system (Luo et al., 2021); hsa-miR-146a-5p, hsa-miR34b-3p, hsa-miR-28-3p, and hsa-miR-412-5p treat oxidative stress injury of the skin due to photoaging (Xiao et al., 2021); and miR-31-5p treats oxidative stress injury of the motor system (Mas-Bargues et al., 2020).

#### 4.3.3 Exosomes and autophagy

Autophagy is an intracellular metabolic self-degradation process in which cytoplasmic proteins and damaged organelles are degraded by lysosomes. Degradation products, such as membrane lipids, proteins, free fatty acids and amino acids, further play important roles in normal cellular metabolism, energy production and cell survival (Wang et al., 2020b). Exosomes are very closely related to molecular signaling levels and intracellular autophagy during biogenesis. Therefore, in recent years, the exploration of exosomes for the treatment of oxidative stress injury through the regulation of cellular autophagy has been extremely hot, and there are certain research results and research bases that are worthy of continued exploration by many scholars.

#### 4.3.4 Proteomics in exosomes

Proteins have important cellular functions, and an increasing number of researchers are focusing their attention on exosomal proteins. To date, more than 1,000 proteins have been identified in exosomes of MSCs(Zhang et al., 2022). As the most direct and effective manifestation of vital functions, exosomal proteins play an important role in all stages of the development of living organisms (Xing et al., 2020). Several studies have reported the proteomic characterization of extracellular vesicles from MSCs. Kim et al. identified 730 proteins in a proteomic analysis of extracellular vesicles from human bone marrow MSCs. Extracellular vesicle proteins contain markers of MSCs as well as signaling molecules regulating the ability of MSCs to self-renew and differentiate (Kim et al., 2012). La Greca et al. used protein histology analysis to identify 560 proteins from pluripotent stem cell-derived extracellular vesicles. Extracellular vesicles shared 37.32% of their proteins with parental cells and were enriched for molecules related to immunity, extracellular matrix and cell adhesion (La Greca et al., 2018). Otero-Ortega et al. investigated the proteomic characterization of adipose-derived MSC-EVs in an experimental animal model of subcortical stroke in rats. Proteomic analysis identified 2,416 proteins, most of which were associated with brain repair functions (Otero-Ortega et al., 2017). In addition to proteomics, genomics, transcriptomics, metabolomics, and lipidomics may be hotspots and directions for future research in the field of “Extracellular Vesicles in the Treatment of Oxidative Stress Injury".

### 4.4 Advantages and limitations

This study has clear advantages. This bibliometric study is the first to identify and characterize all publications in the Web of Science core set on the study “Extracellular Vesicles in the Treatment of Oxidative Stress Injury”. By analyzing the country distribution, institutional collaboration, author interactions, and keyword clustering, the study summarizes the history and future direction of the research field, allowing scholars to have a clear and preliminary understanding of the basic situation of the field. This study summarizes the research hotspots of EVs in the treatment of oxidative stress-related diseases so that scholars can clearly understand the basic situation of this research field.

Small EVs play an important role in oxidative stress-related diseases. In the study of respiratory diseases, EVs can affect lung epithelial cells by reactive oxidizing substances and proteolytic enzymes released by pulmonary neutrophils and monocytes (Fregonese and Stolk, 2008; Bari et al., 2019; Kim et al., 2019; Li et al., 2019). In addition, EVs can also protect lung epithelial cells by regulating protease/antiprotease balance in pneumonia (Bari et al., 2019). In the circulatory system, there is a great deal of evidence that exosomes have a protective effect in ischemic hearts, which can alleviate myocardial I/R injury, promote cardiac regeneration and angiogenesis and inhibit fibrosis (Chen et al., 2017; Zhang et al., 2023). Extracellular vesicles derived from mesenchymal stem cells have many functions. In terms of antibacterial activity, MSC-EVs contain 46 proteins involved in Gram-negative bacterial reactions, which means that MSC-EVs have strong antibacterial activity (Bari et al., 2019). For example, MSC-EVs promote the phagocytosis and antimicrobial activity of pulmonary neutrophils and monocytes by promoting the synthesis of leukotriene B4 (LTB4) (Hao et al., 2019). LTB4 is a leukocyte activator that enhances phagocytosis and promotes the release of antimicrobial drugs that contribute to bacterial clearance (Mancuso et al., 2010). MRP1 inhibition induced by MSC-EVs results in increased release of LTB4 from alveolar macrophages due to its antibacterial activity, bacterial clearance rate and severity of bacterial pneumonia (Hao et al., 2019).

In the classification of medium-sized EVs, microvesicles belong to one of the more important and hot research topics. Microvesicles are intercellular communication carriers and potential disease biomarkers, and their inclusions provide potential markers for a variety of diseases, which is one of the popular directions of liquid biopsy, especially EV miRNAs, which is currently the focus of scholars’ exploration. Through literature dosage analysis, miRNA expression profiles, miRNA-related drugs, miRNA regulatory pathways, and miRNA function of these miRNAs in microvesicles are promising and worth exploring.

The number of large EVs is small, and their apoptotic bodies are closely associated with the development of oxidative stress in various systemic diseases. The relationship between oxidative stress and apoptosis is interactive. Oxidative stress can induce apoptosis through multiple pathways.

This study also has some weaknesses. Although we used the WoSCC for the bibliometric analysis, there are other public and commercial bibliometric databases, and none of the bibliometric databases are considered superior, with citation data varying widely in each database (Hirsch, 2005). For the analysis of cited articles, the biggest limitation of our study is the inherentization of citation analysis, which is based on the absolute number of citations an article receives. The number of citations is a proxy for impact, but there are many factors that influence citation rates (Brandt et al., 2010; Mishra et al., 2018). For example, this strategy may favor older articles. Journal and author self-citation, incomplete citations, and omission bias can also significantly affect citation rates (Brandt et al., 2019).

## 5 Conclusion

To the best of our knowledge, this study is the first bibliometric analysis of “Extracellular Vesicles in the Treatment of Oxidative Stress Injury” using visualization software and data information mining to obtain the current hotspots and trends in this field and to provide a theoretical basis for its scientific research. The future research hotspots in this field may be “microRNA”, “autophagy”, “histology” and “therapy”. “Relevant researchers can use the results of this study to improve their knowledge and understanding of this field and encourage them to explore further.

## Data Availability

The original contributions presented in the study are included in the article/Supplementary Material, further inquiries can be directed to the corresponding authors.

## References

[B1] Atkin-Smith G. K. Miles M. A. Tixeira R. Lay F. T. Duan M. Hawkins C. J. (2019). Plexin B2 is a regulator of monocyte apoptotic cell disassembly. Cell Rep., 29, 1821–1831. 10.1016/j.celrep.2019.10.014 31722200

[B2] Bari E. Ferrarotti I. Di Silvestre D. Grisoli P. Barzon V. Balderacchi A. (2019). Adipose mesenchymal extracellular vesicles as alpha-1-antitrypsin physiological delivery systems for lung regeneration. Cells, 8, 965. 10.3390/cells8090965 31450843 PMC6770759

[B3] Bisht S. Faiq M. Tolahunase M. Dada R. (2017). Oxidative stress and male infertility. Nat. Rev. Urol., 14, 470–485. 10.1038/nrurol.2017.69 28508879

[B4] Brandt J. S. Downing A. C. Howard D. L. Kofinas J. D. Chasen S. T. (2010). Citation classics in obstetrics and gynecology: the 100 most frequently cited journal articles in the last 50 years. Am. J. Obstet. Gynecol., 203, 355.e1-e7. 10.1016/j.ajog.2010.07.025 20875501

[B5] Brandt J. S. Hadaya O. Schuster M. Rosen T. Sauer M. V. Ananth C. V. (2019). A bibliometric analysis of top-cited journal articles in obstetrics and gynecology. JAMA Netw. Open, 2, e1918007. 10.1001/jamanetworkopen.2019.18007 31860106 PMC6991228

[B6] Buzas E. I. (2023). The roles of extracellular vesicles in the immune system. Nat. Rev. Immunol., 23, 236–250. 10.1038/s41577-022-00763-8 35927511 PMC9361922

[B7] Cao M. Ning J. Hernandez-Lara C. I. Belzile O. Wang Q. Dutcher S. K. (2015). Uni-directional ciliary membrane protein trafficking by a cytoplasmic retrograde IFT motor and ciliary ectosome shedding. Elife, 4, e05242. 10.7554/eLife.05242 25688564 PMC4362204

[B8] Caplan A. I. Correa D. (2011). The MSC: an injury drugstore. Cell Stem Cell, 9, 11–15. 10.1016/j.stem.2011.06.008 21726829 PMC3144500

[B9] Chen G. H. Xu J. Yang Y. J. (2017). Exosomes: promising sacks for treating ischemic heart disease? Am. J. Physiol. Heart Circ. Physiol., 313, H508-h523. 10.1152/ajpheart.00213.2017 28646026

[B10] Chen J. Li C. Liang Z. Li C. Li Y. Zhao Z. (2021). Human mesenchymal stromal cells small extracellular vesicles attenuate sepsis-induced acute lung injury in a mouse model: the role of oxidative stress and the mitogen-activated protein kinase/nuclear factor kappa B pathway. Cytotherapy, 23, 918–930. 10.1016/j.jcyt.2021.05.009 34272174

[B11] Ding D. C. Chang Y. H. Shyu W. C. Lin S. Z. (2015). Human umbilical cord mesenchymal stem cells: a new era for stem cell therapy. Cell Transpl., 24, 339–347. 10.3727/096368915X686841 25622293

[B12] Di Vizio D. Morello M. Dudley A. C. Schow P. W. Adam R. M. Morley S. (2012). Large oncosomes in human prostate cancer tissues and in the circulation of mice with metastatic disease. Am. J. Pathol., 181, 1573–1584. 10.1016/j.ajpath.2012.07.030 23022210 PMC3483805

[B13] Dong X. Tan Y. Zhuang D. Hu T. Zhao M. (2022). Global characteristics and trends in research on ferroptosis: a data-driven bibliometric study. Oxid. Med. Cell Longev., 2022, 8661864. 10.1155/2022/8661864 35087622 PMC8787456

[B14] Fregonese L. Stolk J. (2008). Hereditary alpha-1-antitrypsin deficiency and its clinical consequences. Orphanet J. Rare Dis., 3, 16. 10.1186/1750-1172-3-16 18565211 PMC2441617

[B15] Friedenstein A. J. Chailakhyan R. K. Latsinik N. V. Panasyuk A. F. Keiliss-Borok I. V. (1974). Stromal cells responsible for transferring the microenvironment of the hemopoietic tissues. Cloning *in vitro* and retransplantation *in vivo* . Transplantation, 17, 331–340. 10.1097/00007890-197404000-00001 4150881

[B16] Hao Q. Gudapati V. Monsel A. Park J. H. Hu S. Kato H. (2019). Mesenchymal stem cell-derived extracellular vesicles decrease lung injury in mice. J. Immunol., 203, 1961–1972. 10.4049/jimmunol.1801534 31451675 PMC6760999

[B17] Hirsch J. E. (2005). An index to quantify an individual's scientific research output. Proc. Natl. Acad. Sci. U. S. A., 102, 16569–16572. 10.1073/pnas.0507655102 16275915 PMC1283832

[B18] Kalra H. Simpson R. J. Ji H. Aikawa E. Altevogt P. Askenase P. (2012). Vesiclepedia: a compendium for extracellular vesicles with continuous community annotation. PLoS Biol., 10, e1001450. 10.1371/journal.pbio.1001450 23271954 PMC3525526

[B19] Kerviel A. Zhang M. Altan-Bonnet N. (2021). A new infectious unit: extracellular vesicles carrying virus populations. Annu. Rev. Cell Dev. Biol., 37, 171–197. 10.1146/annurev-cellbio-040621-032416 34270326

[B20] Kim H. R. Mun Y. Lee K. S. Park Y. J. Park J. S. Park J. H. (2018). T-cell microvilli constitute immunological synaptosomes that carry messages to antigen-presenting cells. Nat. Commun., 9, 3630. 10.1038/s41467-018-06090-8 30194420 PMC6128830

[B21] Kim H. S. Choi D. Y. Yun S. J. Choi S. M. Kang J. W. Jung J. W. (2012). Proteomic analysis of microvesicles derived from human mesenchymal stem cells. J. Proteome Res., 11, 839–849. 10.1021/pr200682z 22148876

[B22] Kim S. Y. Joglekar M. V. Hardikar A. A. Phan T. H. Khanal D. Tharkar P. (2019). Placenta stem/stromal cell-derived extracellular vesicles for potential use in lung repair. Proteomics, 19, e1800166. 10.1002/pmic.201800166 31318160

[B23] La Greca A. Solari C. Furmento V. Lombardi A. Biani M. C. Aban C. (2018). Extracellular vesicles from pluripotent stem cell-derived mesenchymal stem cells acquire a stromal modulatory proteomic pattern during differentiation. Exp. Mol. Med., 50 (9), 1–12. 10.1038/s12276-018-0142-x PMC6131549 30201949

[B24] Li J. W. Wei L. Han Z. Chen Z. (2019). Mesenchymal stromal cells-derived exosomes alleviate ischemia/reperfusion injury in mouse lung by transporting anti-apoptotic miR-21-5p. Eur. J. Pharmacol., 852, 68–76. 10.1016/j.ejphar.2019.01.022 30682335

[B25] Liu C. Su W. Tan Z. Zhang J. Dong W. (2023). The interaction between microbiota and immune in intestinal inflammatory diseases: global research status and trends. Front. Cell Infect. Microbiol., 13, 1128249. 10.3389/fcimb.2023.1128249 36824689 PMC9941562

[B26] Liu H. Li R. Liu T. Yang L. Yin G. Xie Q. (2020). Immunomodulatory effects of mesenchymal stem cells and mesenchymal stem cell-derived extracellular vesicles in rheumatoid arthritis. Front. Immunol., 11, 1912. 10.3389/fimmu.2020.01912 32973792 PMC7468450

[B27] Luo Q. Xian P. Wang T. Wu S. Sun T. Wang W. (2021). Antioxidant activity of mesenchymal stem cell-derived extracellular vesicles restores hippocampal neurons following seizure damage. Theranostics, 11, 5986–6005. 10.7150/thno.58632 33897894 PMC8058724

[B28] Ma L. Li Y. Peng J. Wu D. Zhao X. Cui Y. (2015). Discovery of the migrasome, an organelle mediating release of cytoplasmic contents during cell migration. Cell Res., 25, 24–38. 10.1038/cr.2014.135 25342562 PMC4650581

[B29] Mancuso P. Lewis C. Serezani C. H. Goel D. Peters-Golden M. (2010). Intrapulmonary administration of leukotriene B4 enhances pulmonary host defense against pneumococcal pneumonia. Infect. Immun., 78, 2264–2271. 10.1128/IAI.01323-09 20231413 PMC2863513

[B30] Marki A. Buscher K. Lorenzini C. Meyer M. Saigusa R. Fan Z. (2021). Elongated neutrophil-derived structures are blood-borne microparticles formed by rolling neutrophils during sepsis. J. Exp. Med., 218, e20200551. 10.1084/jem.20200551 33275138 PMC7721910

[B31] Mas-Bargues C. Sanz-Ros J. RomáN-DomíNGUEZ A. Gimeno-Mallench L. IngléS M. ViñA J. (2020). Extracellular vesicles from healthy cells improves cell function and stemness in premature senescent stem cells by miR-302b and HIF-1α activation. Biomolecules, 10, 957. 10.3390/biom10060957 32630449 PMC7357081

[B32] Mathieu M. NéVO N. Jouve M. Valenzuela J. I. Maurin M. Verweij F. J. (2021). Specificities of exosome versus small ectosome secretion revealed by live intracellular tracking of CD63 and CD9. Nat. Commun., 12, 4389. 10.1038/s41467-021-24384-2 34282141 PMC8289845

[B33] Melentijevic I. Toth M. L. Arnold M. L. Guasp R. J. Harinath G. Nguyen K. C. (2017). *C. elegans* neurons jettison protein aggregates and mitochondria under neurotoxic stress. Nature, 542, 367–371. 10.1038/nature21362 28178240 PMC5336134

[B34] Mishra S. Fegley B. D. Diesner J. Torvik V. I. (2018). Self-citation is the hallmark of productive authors, of any gender. PLoS One, 13, e0195773. 10.1371/journal.pone.0195773 30256792 PMC6157831

[B35] Morrison T. J. Jackson M. V. Cunningham E. K. Kissenpfennig A. Mcauley D. F. O'Kane C. M. (2017). Mesenchymal stromal cells modulate macrophages in clinically relevant lung injury models by extracellular vesicle mitochondrial transfer. Am. J. Respir. Crit. Care Med., 196, 1275–1286. 10.1164/rccm.201701-0170OC 28598224 PMC5694830

[B36] Nojehdehi S. Soudi S. Hesampour A. Rasouli S. Soleimani M. Hashemi S. M. (2018). Immunomodulatory effects of mesenchymal stem cell-derived exosomes on experimental type-1 autoimmune diabetes. J. Cell Biochem., 119, 9433–9443. 10.1002/jcb.27260 30074271

[B37] Ogisu K. Fujio M. Tsuchiya S. Tsuboi M. Qi C. Toyama N. (2020). Conditioned media from mesenchymal stromal cells and periodontal ligament fibroblasts under cyclic stretch stimulation promote bone healing in mouse calvarial defects. Cytotherapy, 22, 543–551. 10.1016/j.jcyt.2020.05.008 32798177

[B38] Otero-Ortega L. Laso-GarcíA F. GóMEZ-De Frutos M. D. RodríGUEZ-Frutos B. Pascual-Guerra J. Fuentes B. (2017). White matter repair after extracellular vesicles administration in an experimental animal model of subcortical stroke. Sci. Rep., 7, 44433. 10.1038/srep44433 28300134 PMC5353554

[B39] Qin S. B. Peng D. Y. Lu J. M. Ke Z. P. (2018). MiR-182-5p inhibited oxidative stress and apoptosis triggered by oxidized low-density lipoprotein via targeting toll-like receptor 4. J. Cell Physiol., 233, 6630–6637. 10.1002/jcp.26389 29226948

[B40] Raffaghello L. Bianchi G. Bertolotto M. Montecucco F. Busca A. Dallegri F. (2008). Human mesenchymal stem cells inhibit neutrophil apoptosis: a model for neutrophil preservation in the bone marrow niche. Stem Cells, 26, 151–162. 10.1634/stemcells.2007-0416 17932421

[B41] Sies H. (2015). Oxidative stress: a concept in redox biology and medicine. Redox Biol., 4, 180–183. 10.1016/j.redox.2015.01.002 25588755 PMC4309861

[B42] Wang Z. G. He Z. Y. Liang S. Yang Q. Cheng P. Chen A. M. (2020b). Comprehensive proteomic analysis of exosomes derived from human bone marrow, adipose tissue, and umbilical cord mesenchymal stem cells. Stem Cell Res. Ther., 11, 511. 10.1186/s13287-020-02032-8 33246507 PMC7694919

[B43] Wang T. Jian Z. Baskys A. Yang J. Li J. Guo H. (2020a). MSC-derived exosomes protect against oxidative stress-induced skin injury via adaptive regulation of the NRF2 defense system. Biomaterials, 257, 120264. 10.1016/j.biomaterials.2020.120264 32791387

[B44] Xia C. Zeng Z. Fang B. Tao M. Gu C. Zheng L. (2019). Mesenchymal stem cell-derived exosomes ameliorate intervertebral disc degeneration via antioxidant and anti-inflammatory effects. Free Radic. Biol. Med., 143, 1–15. 10.1016/j.freeradbiomed.2019.07.026 31351174

[B45] Xiao X. Xu M. Yu H. Wang L. Li X. Rak J. (2021). Mesenchymal stem cell-derived small extracellular vesicles mitigate oxidative stress-induced senescence in endothelial cells via regulation of miR-146a/Src. Signal Transduct. Target Ther., 6, 354. 10.1038/s41392-021-00765-3 34675187 PMC8531331

[B46] Xing X. Han S. Cheng G. Ni Y. Li Z. Li Z. (2020). Proteomic analysis of exosomes from adipose-derived mesenchymal stem cells: a novel therapeutic strategy for tissue injury. Biomed. Res. Int., 2020, 6094562. 10.1155/2020/6094562 32190672 PMC7073480

[B47] Zhang W. Wang T. Xue Y. Zhan B. Lai Z. Huang W. (2023). Research progress of extracellular vesicles and exosomes derived from mesenchymal stem cells in the treatment of oxidative stress-related diseases. Front. Immunol., 14, 1238789. 10.3389/fimmu.2023.1238789 37646039 PMC10461809

[B48] Zhang Z. Mi T. Jin L. Li M. Zhanghuang C. Wang J. (2022). Comprehensive proteomic analysis of exosome mimetic vesicles and exosomes derived from human umbilical cord mesenchymal stem cells. Stem Cell Res. Ther., 13, 312. 10.1186/s13287-022-03008-6 35841000 PMC9284776

[B49] Zhao R. Wang L. Wang T. Xian P. Wang H. Long Q. (2022). Inhalation of MSC-EVs is a noninvasive strategy for ameliorating acute lung injury. J. Control Release, 345, 214–230. 10.1016/j.jconrel.2022.03.025 35307508

