# Supplementary material for: Extracellular vesicles in the treatment of oxidative stress injury: global research status and trends
Source: Front Mol Biosci. 2024 Feb 15;10:1273113. doi: 10.3389/fmolb.2023.1273113 (PMC10903538; doi:10.3389/fmolb.2023.1273113)
Supplement: Supplementary file 1 [file Table1.docx]

| organization（country） | Article Counts | Total number of citations | Average citations | Total number of first authors | Number of first author citations | Average number of first author citations |
| --- | --- | --- | --- | --- | --- | --- |
| Kaohsiung Chang Gung Mem Hosp（Taiwan, China） | 48 | 477 | 9.94 | 6 | 50 | 8.33 |
| Jiangsu Univ（China） | 20 | 434 | 21.70 | 8 | 129 | 16.13 |
| Shanghai Jiao Tong Univ（China） | 68 | 292 | 4.29 | 29 | 82 | 2.83 |
| Univ Med Ctr Utrecht（Netherlands） | 5 | 279 | 55.80 | 3 | 95 | 31.67 |
| ASTAR（Singapore） | 2 | 184 | 92.00 | 0 | 0 | 0.00 |
| Univ Valencia（Spain） | 16 | 165 | 10.31 | 6 | 17 | 2.83 |
| Univ Texas Med Branch（the United States） | 33 | 148 | 4.48 | 19 | 90 | 4.74 |
| Natl Univ Singapore（Singapore） | 4 | 134 | 33.50 | 2 | 39 | 19.50 |
| Univ Pittsburgh  （the United States） | 18 | 132 | 7.33 | 4 | 3 | 0.75 |
| Univ Calif San Francisco  （the United States） | 14 | 111 | 7.93 | 3 | 29 | 9.67 |

Table 1 Top 10 Research Institutions

Table 2 Introduction to Publications

| Journal | [Article Counts](javascript:;) | Total Citations | Average Citations | IF |
| --- | --- | --- | --- | --- |
| Stem Cell Research & Therapy | 38 | 206 | 5.42 | 7.5 |
| PLoS One | 18 | 168 | 9.88 | 3.7 |
| Stem Cell Research | 1 | 92 | 92.00 | 1.2 |
| International Journal of Molecular Sciences | 59 | 72 | 1.22 | 5.6 |
| Journal of Cellular and Molecular Medicine | 13 | 70 | 5.38 | 5.3 |
| Scientific Reports | 20 | 66 | 3.03 | 4.6 |
| International Journal of Cardiology | 3 | 63 | 31.00 | 3.5 |
| Oxidative Medicine and Cellular Longevity | 38 | 62 | 1.63 | 7.31 |
| Free Radical Biology and Medicine | 38 | 62 | 1.63 | 7.4 |
| Stem Cells International | 12 | 57 | 4.75 | 4.3 |

Table 3 Frequency of commonly used keywords

| Rank | Keywords | Count | Year | Rank | Keywords | Count | Year |
| --- | --- | --- | --- | --- | --- | --- | --- |
| 1 | \| oxidative stress \| \| --- \| | 657 | 2001 | 26 | \| gene expression \| \| --- \| | 43 | 2014 |
| 2 | \| extracellular vesicles \| \| --- \| | 410 | 2014 | 27 | \| pathway \| \| --- \| | 41 | 2014 |
| 3 | \| exosome \| \| --- \| | 242 | 2008 | 28 | \| disease \| \| --- \| | 40 | 2014 |
| 4 | \| expression \| \| --- \| | 170 | 2007 | 29 | \| myocardial infarction \| \| --- \| | 40 | 2016 |
| 5 | \| mesenchymal stem cells \| \| --- \| | 142 | 2016 | 30 | \| differentiation \| \| --- \| | 39 | 2013 |
| 6 | \| cells \| \| --- \| | 107 | 2014 | 31 | \| protein \| \| --- \| | 39 | 2007 |
| 7 | apoptosis | 101 | 2010 | 32 | \| inhibition \| \| --- \| | 37 | 2017 |
| 8 | activation | 92 | 2007 | 33 | \| secretion \| \| --- \| | 37 | 2016 |
| 9 | \| stem cells \| \| --- \| | 89 | 2015 | 34 | \| autophagy \| \| --- \| | 36 | 2014 |
| 10 | \| inflammation \| \| --- \| | 89 | 2016 | 35 | \| biomarkers \| \| --- \| | 35 | 2013 |
| 11 | \| microvesicles \| \| --- \| | 70 | 2013 | 36 | \| bone marrow \| \| --- \| | 35 | 2014 |
| 12 | \| stromal cells \| \| --- \| | 76 | 2015 | 37 | \| parkinsons disease \| \| --- \| | 34 | 2008 |
| 13 | \| in vitro \| \| --- \| | 76 | 2012 | 38 | \| repair \| \| --- \| | 34 | 2018 |
| 14 | \| injury \| \| --- \| | 69 | 2017 | 39 | \| growth \| \| --- \| | 30 | 2005 |
| 15 | \| mechanisms \| \| --- \| | 63 | 2013 | 40 | \| pathogenesis \| \| --- \| | 30 | 2010 |
| 16 | \| proliferation \| \| --- \| | 58 | 2016 | 41 | \| ischemia reperfusion injury \| \| --- \| | 29 | 2016 |
| 17 | \| microRNAs \| \| --- \| | 57 | 2016 | 42 | \| adipose tissue \| \| --- \| | 28 | 2007 |
| 18 | \| Alzheimer’s disease \| \| --- \| | 55 | 2010 | 43 | \| transplantation \| \| --- \| | 28 | 2019 |
| 19 | \| angiogenesis \| \| --- \| | 55 | 2017 | 44 | \| macrophages \| \| --- \| | 27 | 2019 |
| 20 | \| Oxidative stress \| \| --- \| | 53 | 2016 | 45 | \| nf kappa b \| \| --- \| | 27 | 2012 |
| 21 | \| biogenesis \| \| --- \| | 51 | 2010 | 46 | \| stress \| \| --- \| | 27 | 2008 |
| 22 | \| brain \| \| --- \| | 50 | 2015 | 47 | \| extracellular vesicle \| \| --- \| | 26 | 2018 |
| 23 | \| dysfunction \| \| --- \| | 50 | 2017 | 48 | \| mechanism \| \| --- \| | 26 | 2007 |
| 24 | \| endothelial cells \| \| --- \| | 49 | 2012 | 49 | \| damage \| \| --- \| | 25 | 2019 |
| 25 | \| therapy \| \| --- \| | 44 | 2015 | 50 | \| delivery \| \| --- \| | 25 | 2020 |

Table 4 Keyword co-occurrence network clustering table

| Cluster ID | Size | Mean(Year) | Label (LLR) |
| --- | --- | --- | --- |
| 0 | 94 | 2017 | mesenchymal stem (1426.66, 1.0E-4); cardiovascular disease (948.07, 1.0E-4); stromal cell-derived extracellular vesicle (677.09, 1.0E-4); chronic kidney disease (574.2, 1.0E-4); admsc-derived exosome (512.23, 1.0E-4) |
| 1 | 85 | 2018 | aqueous humor (480.75, 1.0E-4); irradiation-induced hematopoietic system injury (339.42, 1.0E-4); mouse serum (339.42, 1.0E-4); total body (339.42, 1.0E-4); systemic environment (339.42, 1.0E-4) |
| 2 | 69 | 2013 | endothelial cell (523.22, 1.0E-4); mesenchymal stem cell (477.68, 1.0E-4); stem cell (447.09, 1.0E-4); therapeutic potential (412.44, 1.0E-4); human umbilical cord (395.42, 1.0E-4) |
| 3 | 69 | 2017 | ischemic stroke (655.71, 1.0E-4); protein aggregation (607.18, 1.0E-4); neural stem (583.6, 1.0E-4); parkinsons disease (580.16, 1.0E-4); neurotrophic factor (555.31, 1.0E-4) |
| 4 | 64 | 2016 | vascular calcification (803.61, 1.0E-4); secretory protein (378.95, 1.0E-4); vascular research (378.95, 1.0E-4); age-associated stroke (372.63, 1.0E-4); human parturition (366.31, 1.0E-4) |
| 5 | 47 | 2017 | noncoding rna (561.54, 1.0E-4); vitro model (463.36, 1.0E-4); systematic mini review (395.83, 1.0E-4); mitochondrial inactivity (391.17, 1.0E-4); obesity-related cardiomyopathy (391.17, 1.0E-4) |
| 6 | 39 | 2013 | therapeutic approaches (435.39, 1.0E-4); myocardial injury (404.77, 1.0E-4); diabetic cardiomyopathy (332.73, 1.0E-4); 3t3-l1 adipocyte (320.6, 1.0E-4); dependent induction (320.6, 1.0E-4) |
| 7 | 25 | 2011 | polycystic ovary syndrome (372.15, 1.0E-4); comparative lipid peroxidation (221.73, 1.0E-4); proline content (221.73, 1.0E-4); antioxidant defense system (221.73, 1.0E-4); rice cultivar (221.73, 1.0E-4) |
| 8 | 19 | 2007 | antioxidant enzyme (114.5, 1.0E-4); growing rice seedling (114.5, 1.0E-4); lipid peroxidation superoxide anion generation (114.5, 1.0E-4); neuronal firing rate signal transduction (100.12, 1.0E-4); oligodendroglial exosome (100.12, 1.0E-4) |
| 9 | 18 | 2012 | endosome network (300.24, 1.0E-4); blood-borne macrophage-neural cell interaction (300.24, 1.0E-4); cell-based nanozyme brain delivery (300.24, 1.0E-4); pigmented cell (294.61, 1.0E-4); melanosome autophagy (294.61, 1.0E-4) |
| 10 | 11 | 2012 | oligomannose-carrying glycoprotein act (52, 1.0E-4); glia-derived exosome (52, 1.0E-4); oligomannose-binding lectin (52, 1.0E-4); neurite outgrowth (52, 1.0E-4); neuronal survival (52, 1.0E-4) |
| 11 | 8 | 2012 | disease (21.06, 1.0E-4); retinal function (21.06, 1.0E-4); alpha-crystallin (21.06, 1.0E-4); novel role (21.06, 1.0E-4); extracellular vesicle (0.39, 1.0) |
