# Supplementary material for: Extracellular vesicles in the treatment of oxidative stress injury: global research status and trends
Source: Front Mol Biosci. 2024 Feb 15;10:1273113. doi: 10.3389/fmolb.2023.1273113 (PMC10903538; doi:10.3389/fmolb.2023.1273113)
Supplement: Supplementary file 2 [file DataSheet1.docx]

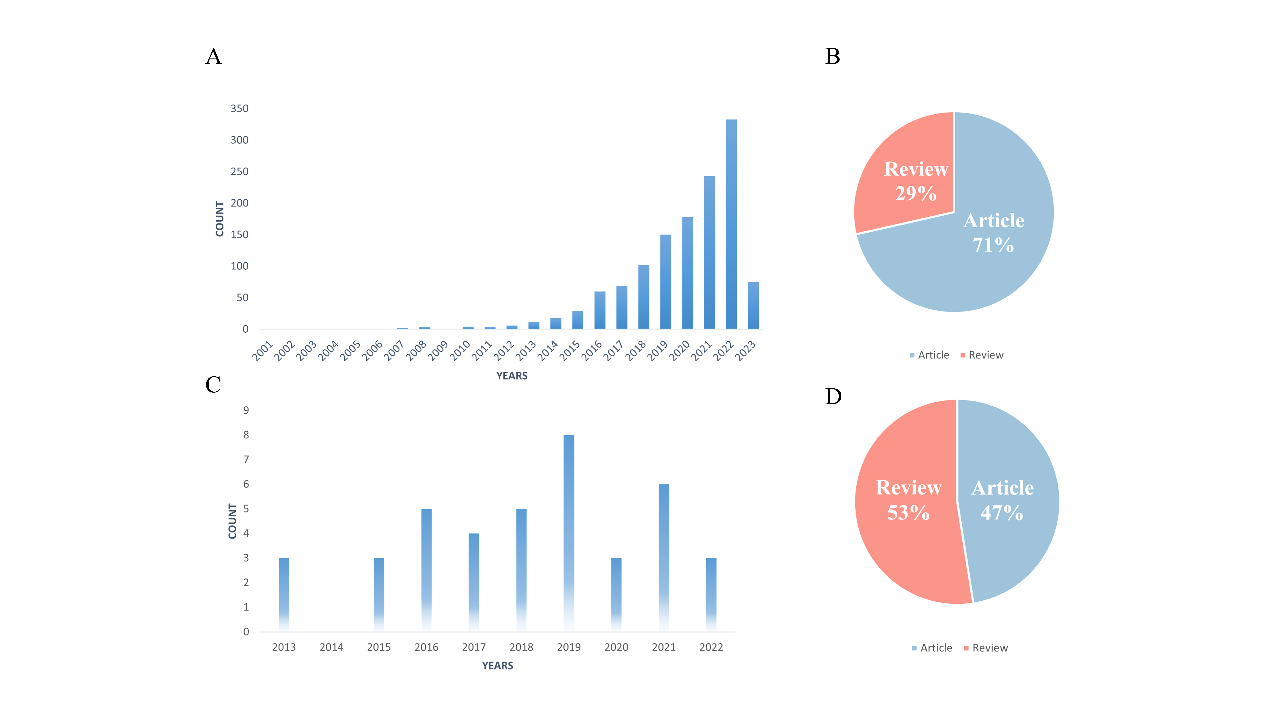


Figure 1 Number of Publications per Year (A, C); Literature Type (B, D); (A, B: All Related Literature; C, D: Highly Cited Essentials).


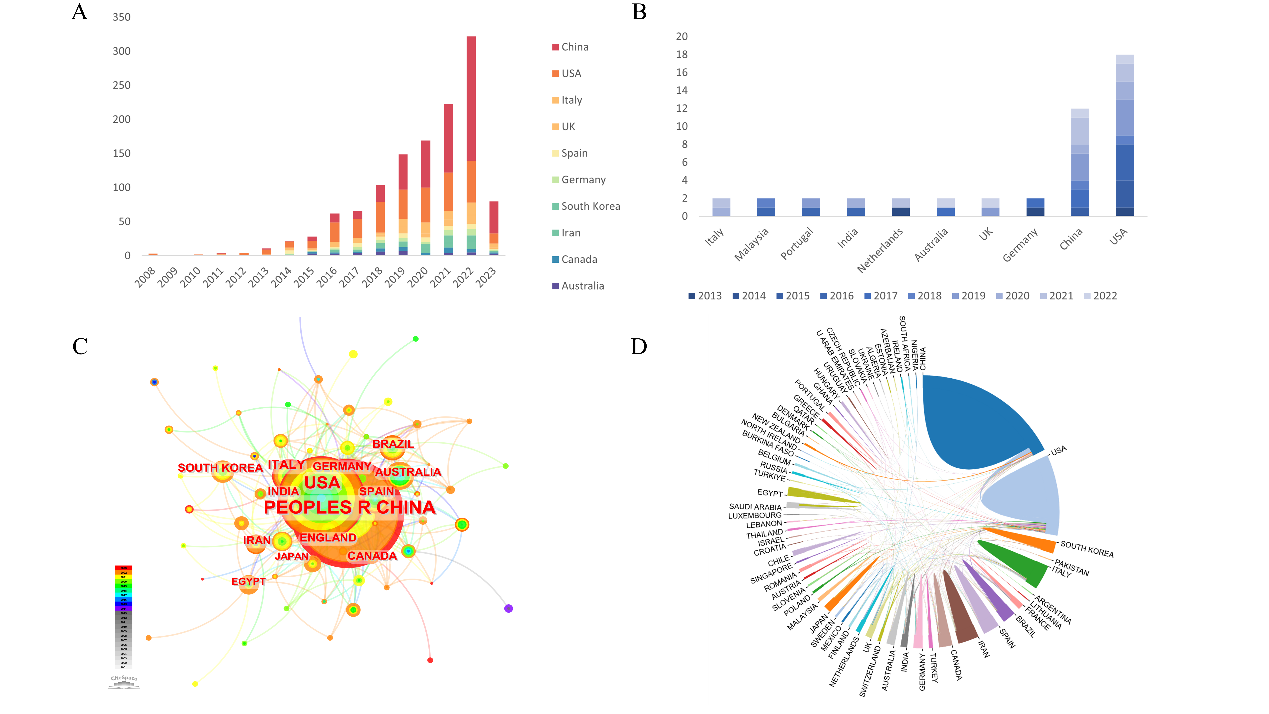


Figure 2 Country of Related Literature (A, B); Country Cooperation (C, D); (A, C, D: All relevant literature; B: Highly cited essence).


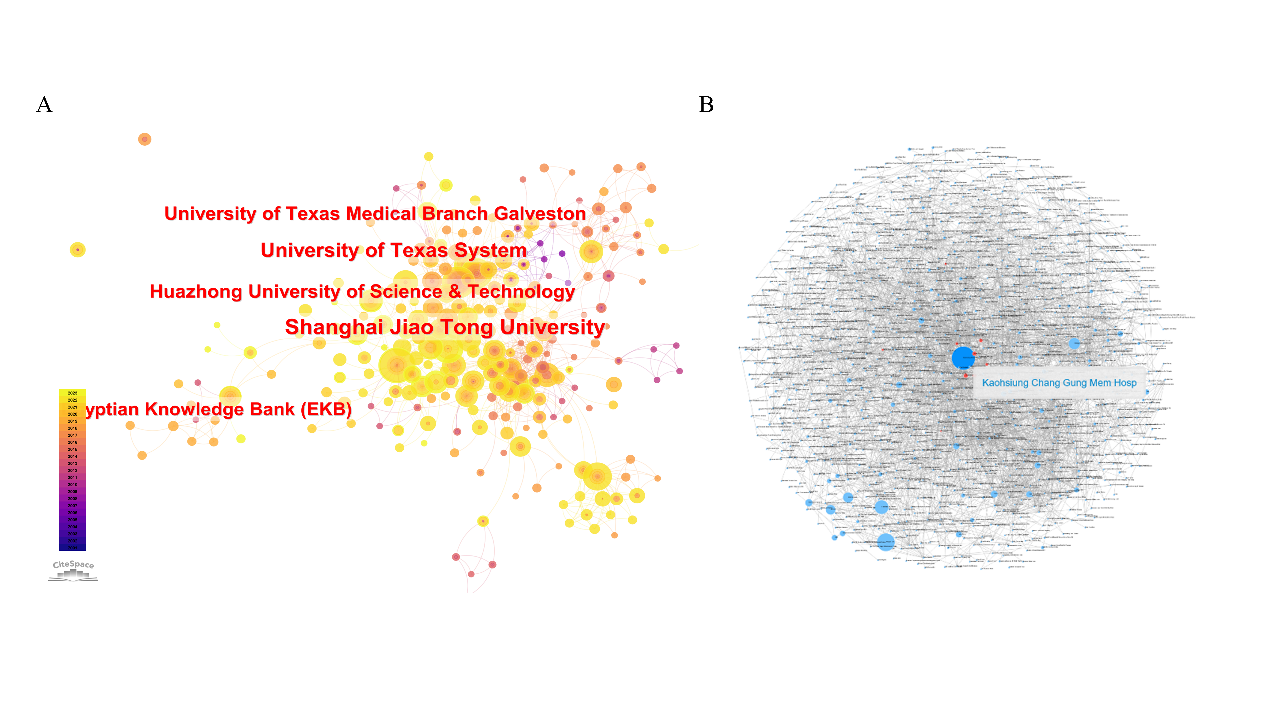


Figure 3 Institutions of relevant literature (A). Cooperation between institutions (B). B: Each small blue dot represents an organization link represents collaboration. The larger the blue dot, the more collaboration. Deep Blue Dot is the agency that cooperates the most. Red Dot is the agency that cooperates with this agency.


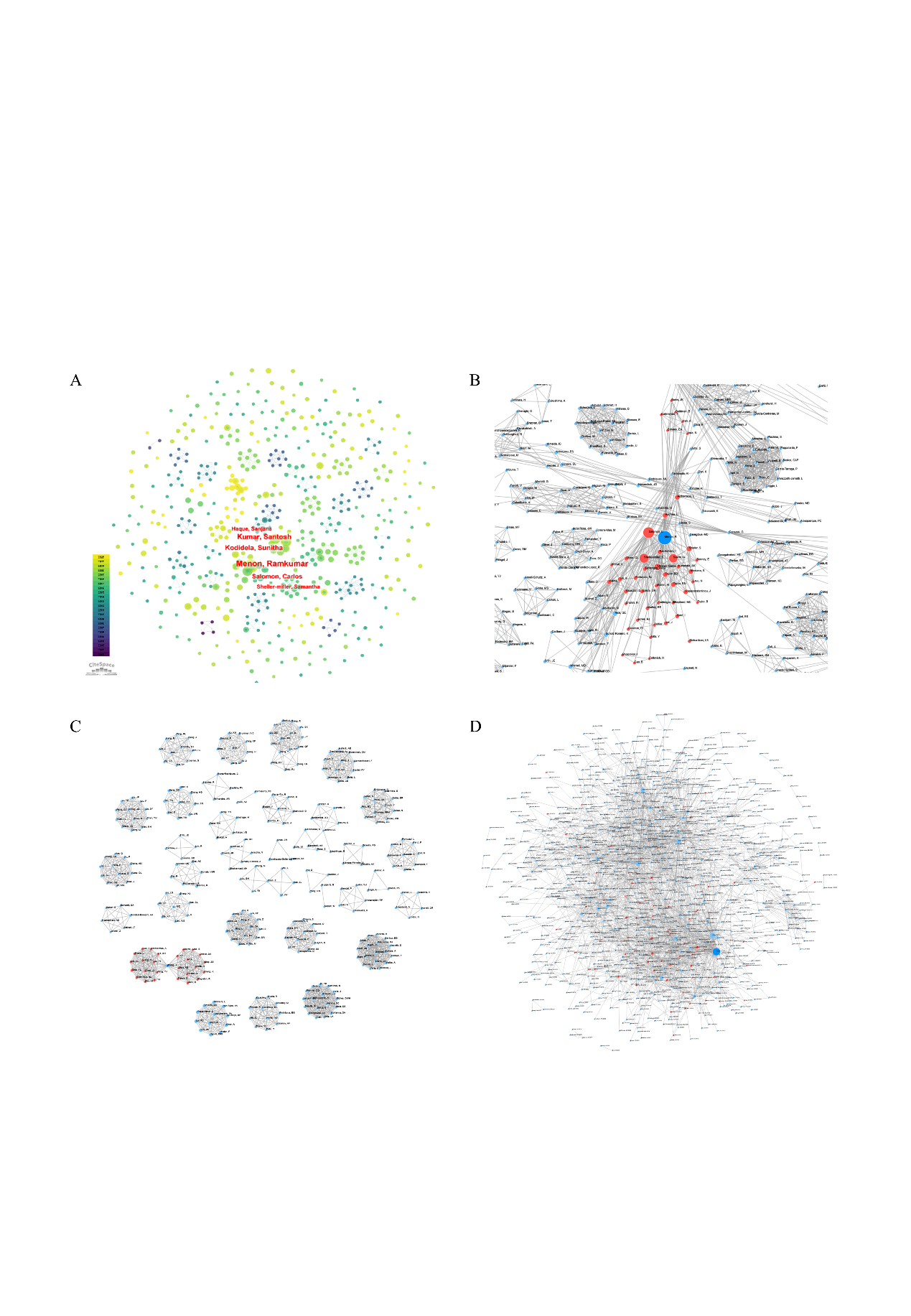


Figure 4 Collaborative relationship between authors of related literature (A, B, C). (A, B: All relevant literature; C: Highly cited essentials; B, C: Each small blue dot represents an author link for collaboration; The bigger the blue dot, the more collaboration; The deep blue dot is the author who collaborates the most; The red dot is the author who collaborates with the author). Citation Relation Network of Journals and Related Documents (D). (D: All relevant literature; Each small blue dot represents an article. Link represents a reference. The larger the blue dot, the more references. The deep blue dot is the most cited article, and the red dot is the article that quoted the article.)


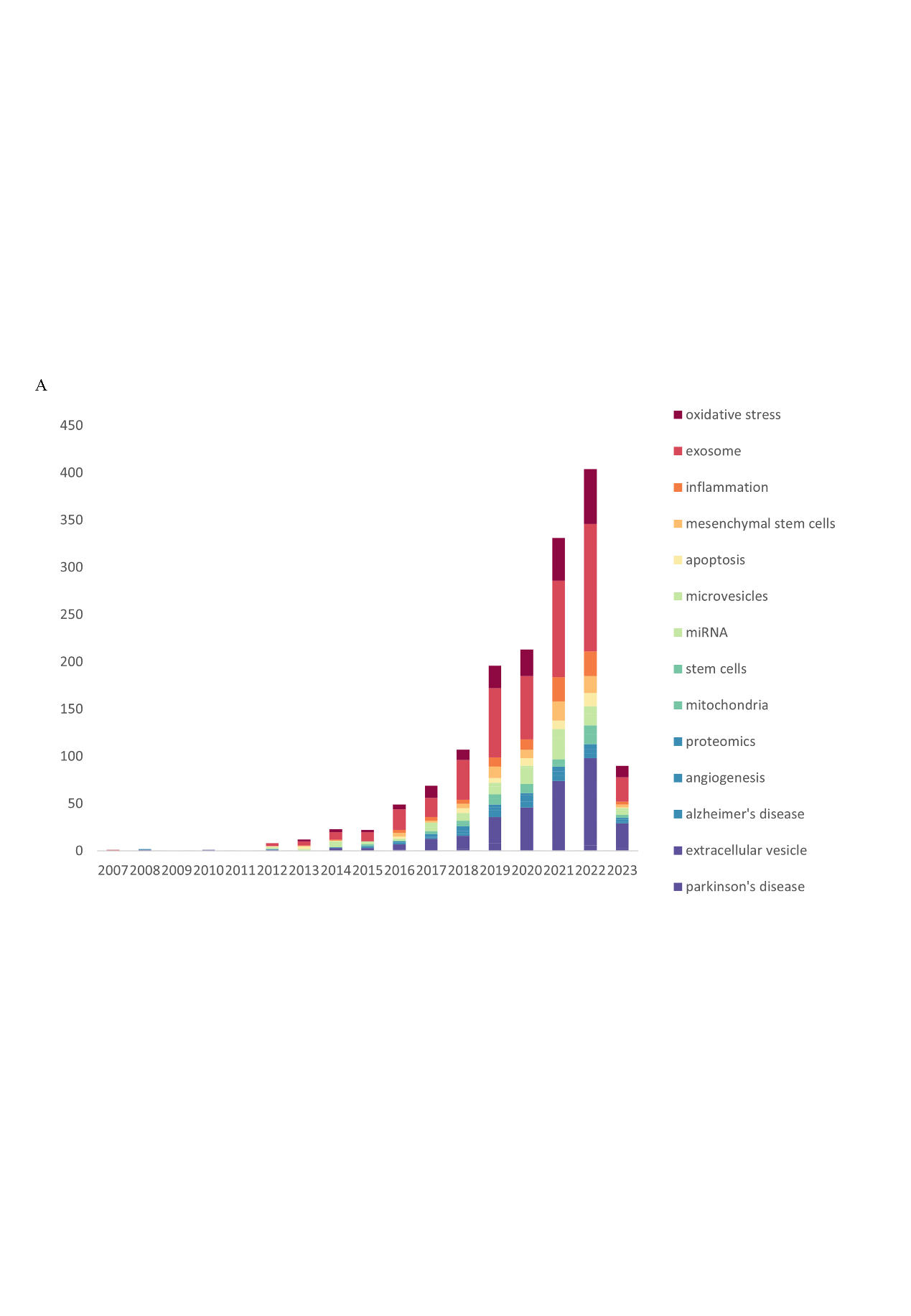


Figure 5 A: Annual number of commonly used keywords.


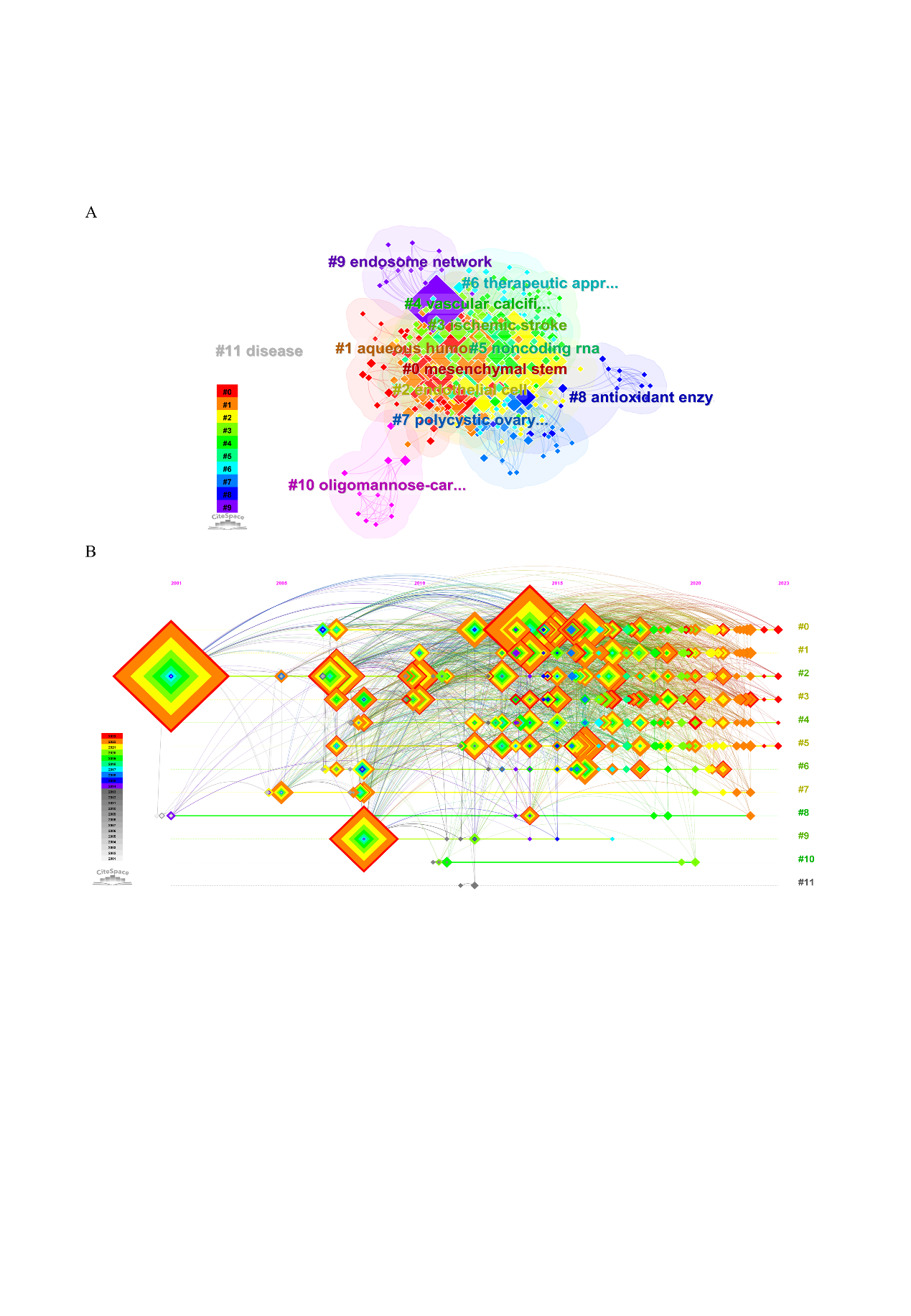


Figure 6 Clustering view of related literature keywords (A); Keyword Clustering Timeline View of Related Literature (B), (A, B: All relevant literature).


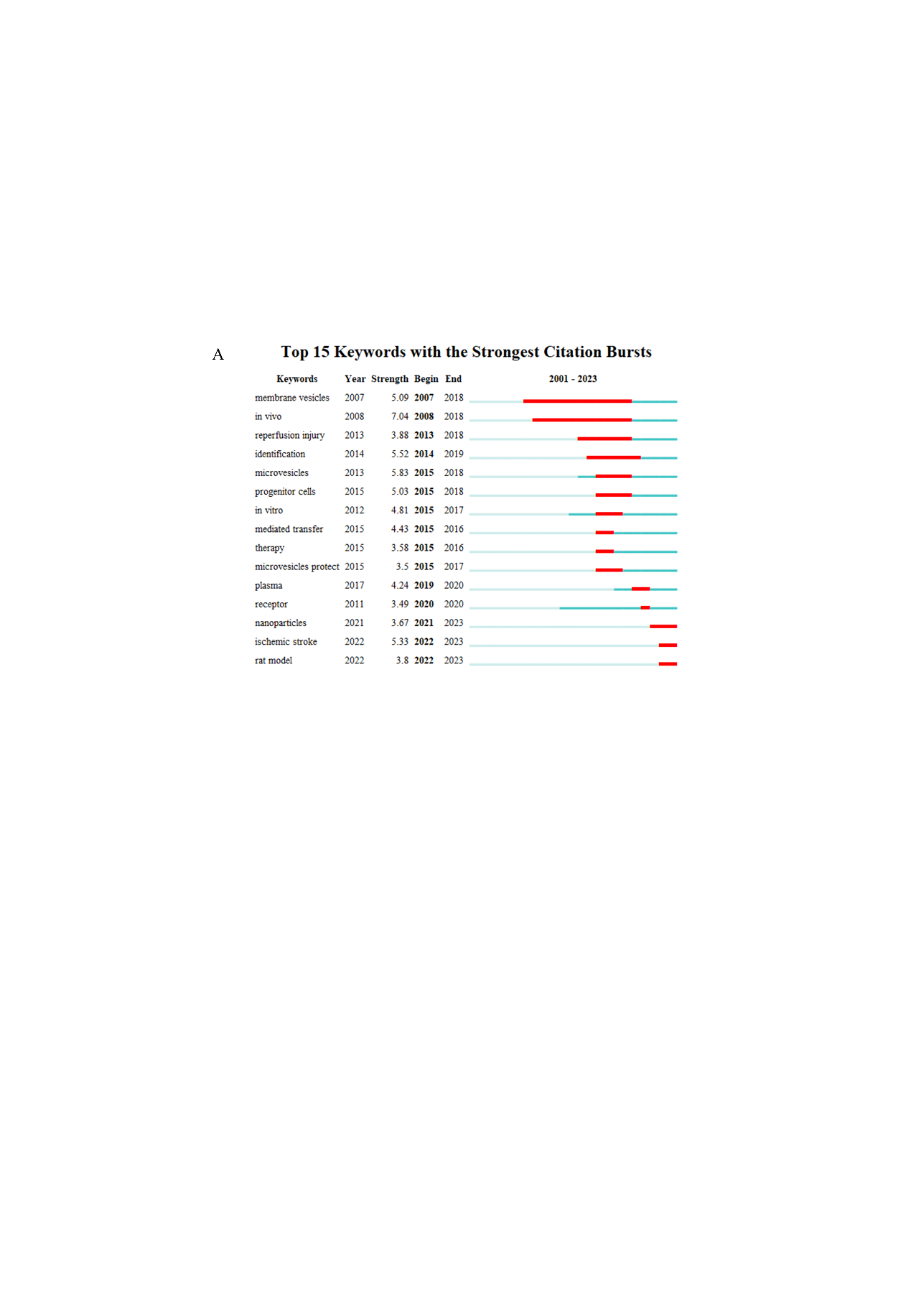


Figure 7 The detonation degree γ [0, 1] = 0.9, Minimum Duration=1.


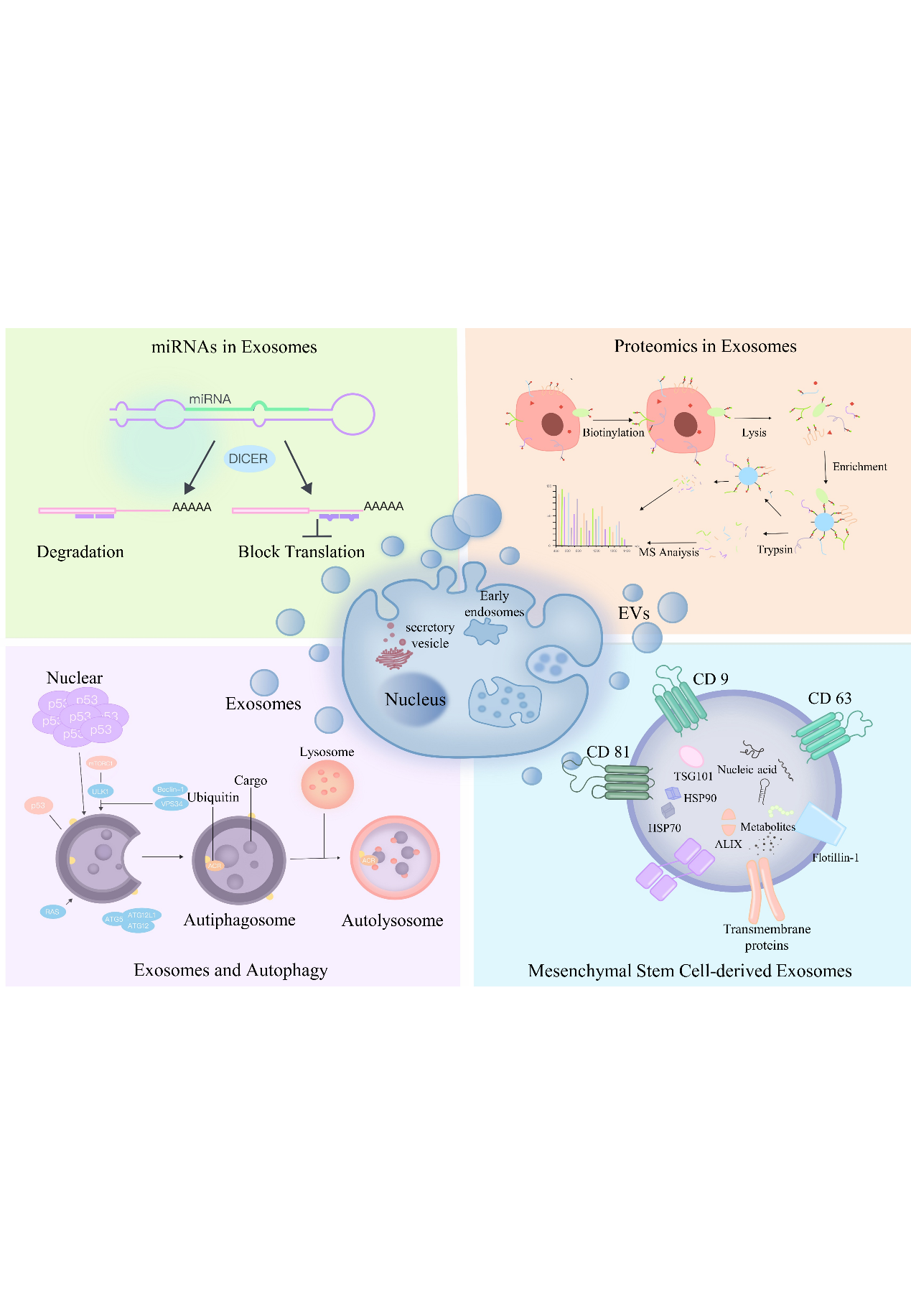


Figure 8 We found out four hot spots in exosomes at present by bibliometrics: Upper left corner: miRNA in exosomes; Upper right corner: exosomes are combined with proteomics; Lower left corner: exosomes and autophagy; Lower right corner: exosomes of mesenchymal stem cells.
